# Anti-plasmodial action of *de novo*-designed, cationic, lysine-branched, amphipathic, helical peptides

**DOI:** 10.1186/1475-2875-11-256

**Published:** 2012-08-01

**Authors:** Naveen K Kaushik, Jyotsna Sharma, Dinkar Sahal

**Affiliations:** 1 Malaria Research Group, International Centre for Genetic Engineering and Biotechnology, Aruna Asaf Ali Marg, New Delhi 110067, India

**Keywords:** Anomalous egress, Anti-plasmodial peptides, *De novo* peptide design, Kinetics of peptide uptake, Peptide binding to DNA, *Plasmodium falciparum*

## Abstract

**Background:**

A lack of vaccine and rampant drug resistance demands new anti-malarials.

**Methods:**

*In vitro* blood stage anti-plasmodial properties of several *de novo*-designed, chemically synthesized, cationic, amphipathic, helical, antibiotic peptides were examined against *Plasmodium falciparum* using SYBR Green assay. Mechanistic details of anti-plasmodial action were examined by optical/fluorescence microscopy and FACS analysis.

**Results:**

Unlike the monomeric decapeptides {(Ac-GXRKXHKXWA-NH_2_) (X = F,ΔF) (Fm_,_ ΔFm IC_50_ >100 μM)}, the lysine-branched,dimeric versions showed far greater potency {IC_50_ (μM) Fd 1.5 , ΔFd 1.39}. The more helical and proteolytically stable ΔFd was studied for mechanistic details. ΔFq, a K-K_2_ dendrimer of ΔFm and (ΔFm)_2_ a linear dimer of ΔFm showed IC_50_ (μM) of 0.25 and 2.4 respectively. The healthy/infected red cell selectivity indices were >35 (ΔFd), >20 (ΔFm)_2_ and 10 (ΔFq). FITC-ΔFd showed rapid and selective accumulation in parasitized red cells. Overlaying DAPI and FITC florescence suggested that ΔFd binds DNA. Trophozoites and schizonts incubated with ΔFd (2.5 μM) egressed anomalously and Band-3 immunostaining revealed them not to be associated with RBC membrane. Prematurely egressed merozoites from peptide-treated cultures were found to be invasion incompetent.

**Conclusion:**

Good selectivity (>35), good resistance index (1.1) and low cytotoxicity indicate the promise of ΔFd against malaria.

## Background

The devastating diseases caused by protozoan parasites are a major burden of the tropics, and in particular, *Plasmodium falciparum,* the causative agent of falciparum malaria, creates a serious public health problem in many areas of the densely populated developing world. The widespread resistance of *P. falciparum* to chloroquine (CQ), which has spread from Asia to Africa, has rendered the drug ineffective against the most dangerous *Plasmodium* strain in many affected regions of the world. Unfortunately, CQ-resistance is associated with cross-resistance to other quinoline drugs, such as quinine and amodiaquine [1]. *Plasmodium falciparum* is genetically diverse and has multiple independent origins of mutations in genes that confer resistance to widely used anti-malarial drugs [2]. Left with just artemisinin to fight against malaria, Arata Kochi, Director of the Malaria Division at the World Health Organization, had felt compelled to say “if we lose artemisinin, we will no longer have an effective cure for malaria” [3]. However, most recently, alarming signs of clinical resistance against artemisinin, in the form of delayed parasite clearance, are being observed in the border between Cambodia and Thailand [4,5]. The challenge of designing an effective vaccine along traditional lines against malaria is that many *P. falciparum* proteins are highly polymorphic and their functions are redundant [6]. More than 200 million new malaria cases reported annually is a challenge [7] that underscores the urgent requirement for new drugs against malaria.

Peptides are an essential component of defence mechanism of all life forms and anti-microbial peptides are evolutionarily ancient biological weapons. Their widespread distribution throughout the living kingdom suggests that anti-microbial peptides may have served a fundamental role in the successful evolution of complex multi-cellular organisms [8]. Despite their ancient lineage, anti-microbial peptides have remained effective defensive weapons, defeating the general belief that bacteria, fungi and viruses can and will develop resistance to any conceivable substance. Among other differences, uniquely anionic charge on bacterial surface is a curious feature that distinguishes the prokaryotic bacteria from their eukaryotic counterparts [9]. Anti-microbial peptides gain selectivity from their ability to target this previously under-appreciated ‘microbial Achille’s heel’ [10-12]. Interestingly, a seminal feature of the malaria parasite-infected red cell is reflected in an altered asymmetry of lipid composition in its cell surface membrane. In contrast to the uninfected, healthy red cell, the malaria-infected red cell shows a translocation of the anionic phosphatidylserine from the inner leaflet to the outer leaflet of the bi-layer [13]. As a result, the FITC-Annexin negative, healthy red cell now turns to become FITC-Annexin positive [14]. Thus a *Plasmodium*-infected red cell seems to mimic the anionic surface charge that characterizes the bacterial cell surface. In principle, this is expected to make malaria-infected red cells become vulnerable to the action of anti-microbial peptides. Indeed, naturally occurring or modified peptides, such as dermaseptin [15], oligoacyllysine [16], cyclosporin A [17], cecropin A [18], NK-2 [14] and meucin [19], have been found to display *in vitro* anti-malarial activity. Some membrane-active, hydrophobic peptides of fungal origin have also been found to exhibit *in vitro* anti-malarial action [20]. However, many of these naturally occurring peptides suffer from drawbacks such as poor potency, stability and selectivity [21]. Therefore, in a bid to improve their performance, efforts are being made to engineer peptides in diverse ways with the aim of reducing their size, improving their stability against proteases and enhancing their selectivity [22-24]. The structure activity relationships of a series of *de novo*-designed, conformationally-constrained helical, amphipathic, cationic peptides against bacteria have earlier been reported [25]. In the present work, the potent anti-plasmodial action of these peptides against both CQ-sensitive and CQ-resistant strains of *P. falciparum* are being reported. The results indicate that a lysine-branched, dimeric peptide ΔFd, which is highly potent (IC_50_ 1.39 μM) across CQ-sensitive and CQ-resistant strains {Resistance Index (IC_50_ CQ resistant strain/ IC_50_ CQ sensitive strain)1.1} of *P. falciparum*, fairly selective against parasitized red blood cells {Selectivity Index (HC_50_ URBC/IC_50_*P. falciparum*) >35) and fairly non toxic to mammalian HeLa cells (TC_50_ >25 μM), stalls parasite growth by causing arrest of ring stage parasite, anomalous egress of trophozoites and premature egress of schizonts that fail to produce invasion competent merozoites.

## Methods

### Peptides

Peptides ΔFm, (ΔFm)_2_, ΔFd, ΔFq, Fm, Fd, D-Lys- ΔFd, prochitinase and E30 (Table 1) were synthesized by Fmoc chemistry-based, manual, solid-phase synthesis. Didehydrophenylalanine (ΔF) was chosen since it is a conformationally-constrained amino acid residue with a proven reputation to confer helical character to peptides. FITC derivatizations of (ΔFm)_2_ and ΔFd were done after linking aminohexanoic acid to the N terminus. Peptides were purified to >95% homogeneity by RPHPLC and characterized by mass spectroscopy and circular dichroism as described previously [25]. Chromatographic and mass spectral characterization is given as follows: RPHPLC profiles of control peptides prochitinase, E30 and bovine insulin (Additional file 1); ΔFm and ΔFd (Additional file 2) Electro Spray Mass Spectroscopy (ESMS) profiles of prochitinase, E30 and bovine insulin (Additional file 3); ESMS profiles of ΔFm and ΔFd (Additional file 4); MALDI of (ΔFm)_2_ (Additional file 5), RPHPLC and mass spectral data for ΔFq (Additional file 6) and ESMS profiles of Fm, D-Lys- ΔFd and Fd (Additional file 7). Peptides corresponding to prochitinase, E30 (a 30 residues-long peptide from Hepatitis E virus ORF3) (synthesized and characterized in house) and bovine insulin (Sigma) were used as controls in experiments on ΔFd mediated selective haemolysis of infected red cells. FITC-Insulin (Sigma) was used as control in experiments done to study the uptake of FITC tagged (ΔFm)_2_ and ΔFd by *Plasmodium-*infected RBCs.

**Table 1:** ***In-vitro *blood stage antiplasmodial activities, resistance and selectivity indices of peptides against different strains of *P. falciparum***

**Peptides**	**Peptide Sequence and design**	**IC**_**50**_***P. falciparum*(μM)**			**Resistance index**	**HC**_**50 **_**URBC μM**
		**3D7**	**Dd2**	**INDO**	**IC**_**50**_**Dd2/ IC**_**50**_**3D7**	
**Fm**	**Ac-GFRKFHKFWA-NH**_**2**_	**>100**	**>100**		**-**	**>100**
**ΔFm**	**Ac-GΔFRKΔFHKΔFWA-NH**_**2**_	**>100**	**>100**	**-**	**-**	**>100**
**ΔFd**		**1.39 ± 0.1**	**1.6 ± 0.09**	**1.5 ± 0.075**	**1.15**	>50 ( >35)*
D-Lys-ΔFd**		**1.8 ± 0.07**	**-**	**-**	**-**	**>50 (> 27)**
**Fd**		**1.5 ± 0.08**	**-**	**-**	**-**	**>50 (>33)**
**(ΔFm)**_**2**_	**Ac-GΔFRKΔFHKΔFWAAGΔFRKΔFHKΔFWA-NH**_**2**_	**2.4 ± 0.15**	**2.5 ± 0.13**	**-**	**1.04**	**>50 (>20)**
**ΔFq**		**0.25 ±0.02**	**-**	**-**	**-**	**2.5 ±0.13 (10)**
**Prochitinase**	**EEPHKAASAEGKK**	**> 40**	**-**	**-**	**-**	**> 40**
**E30**	**NPPDHSAPLGATRPSAPPLPHVVDLPQLGP**	**> 40**	**-**	**-**	**-**	**> 40**
**Insulin**		**> 40**	**-**	**-**	**-**	**> 40**
**Artemisinin**		**0.015**	**0.016**	**0.015**	**1**	
**Chloroquine**		**0.04**	**0.16**	**0.5**	**4**	

* Hemolytic Selectivity index (HC_50_URBC/ IC_50_*Pf *3D7) is shown in parenthesis, **: K^D^ refers to Lysine of D configuration. Values of standard deviation given as ± are based on three independent observations.

### *In vitro *cultivation of *Plasmodium falciparum *

Chloroquine-sensitive (3D7) and CQ-resistant (Dd2 and INDO) strains of *P. falciparum *were maintained in continuous culture according to the method of Trager and Jensen [26] with minor modifications. Cultures were maintained in fresh group O^+ve^ human erythrocytes suspended at 4% haematocrit in complete medium {16.2 g/L RPMI 1640 containing 25 mM HEPES, 11.11 mM glucose (Gibco), 0.2% sodium bicarbonate (Sigma), 0.5% Albumax I (Gibco), 45 μg/litre hypoxanthine (Sigma) and 50 μg/litre gentamicin (Gibco)} and incubated at 37°C under a gas mixture 5% O_2_, 5% CO_2_, and 90% N_2_. Every day, the spent medium was replaced by fresh complete medium to propagate the culture. For INDO strain in culture medium, albumax was replaced by 10% pooled human serum (Innovative Research) as suggested by MR4 [27]. Parasitaemia was monitored by microscopic examination of Giemsa-stained blood smears. Synchronized ring stage parasite was obtained by 5% sorbitol treatment [28]. Trophozoites and schizont-stage parasites were enriched by using Percoll gradient [29].

### Drug dilutions

Stock solutions of peptides and CQ were prepared in water (milli-Q grade) while artemisinin stock solution was in dimethyl sulphoxide (DMSO). All stocks were then diluted with culture medium to achieve the required drug concentrations. The concentration of peptide solution in water was based on A_280_ [ϵ (M^-1^ cm^-1^) ΔF (didehydrophenylalanine) 19,000, W (Tryptophan) 5,000)]. Thus ϵ_280_ were 62,000, 124,000, 124,000 and 248,000 for ΔFm, (ΔFm)_2_, ΔFd and ΔFq respectively. The concentration of FITC-peptides was based on A_495_ [ϵ(M^-1^ cm^-1^) FITC 77,000 for (ΔFm)_2_ with one FITC and 154,000 for ΔFd with two FITC per molecule]. Drugs and peptides solutions were placed in 96-well flat bottom tissue culture grade plates (Corning).

### Assay for anti-plasmodial activity

For drug screening, SYBR green I based fluorescence assay was used as described previously by Smilkstein *et al.*[30]. Sorbitol synchronized ring stage parasites (haematocrit: 2%, parasitaemia: 1%, 100 μl) under normal culture conditions were incubated in the absence or presence of increasing concentrations of peptides in water. CQ and artemisinin were used as positive controls. Vehicle control 0.4% DMSO (which was found to be non-toxic to parasite) was used in case of artemisinin. After 48 hr of incubation 100 μl of SYBR Green I buffer [0.2 μl of 10,000 X SYBR Green I (Invitrogen) per ml of lysis buffer {Tris (20 mM; pH 7.5), EDTA (5 mM), saponin (0.008%; wt/vol), and Triton X-100 (0.08%; vol/vol)}] was added to each well, mixed twice gently with multi-channel pipette and incubated in dark at 37^0^C for 1 h. Fluorescence was measured with a Victor fluorescence multi-well plate reader (Perkin Elmer) with excitation and emission wavelength centred at 485 and 530 nm, respectively. Fluorescence counts for CQ (0.1 μM for 3D7, 1 μM for INDO) were subtracted from counts in each well. The fluorescence counts were plotted against the drug concentration and IC_50_ (the 50% inhibitory concentration) was determined by analysis of dose–response curves. Results of the above mentioned fluorescence-based assay were validated microscopically by examination of Giemsa-stained smears of peptide-treated parasite cultures. Statistical significance of relative potencies of peptides was determined by student’s *T* test.

### *In vitro* stage dependence of action

Stage specificity of action of ΔFd on the parasite’s blood stage life cycle was determined by microscopic analysis of the effect of ΔFd on each of the three stages (ring, trophozoite and schizont) of the parasite life cycle. Synchronized stages were obtained by sorbitol-mediated synchronization repeated thrice (synchronization 1, medium washed, incubation for 3 hr, 37^0^C, synchronization 2, medium washed and culture allowed to grow in complete medium for 48 hr. At this stage the culture was synchronized a third time to obtain highly synchronized ring stage culture). This culture was grown for 24 hr and 38 hr to obtain trophozoite and schizont stage cultures respectively. Both trophozoite and schizont enriched cultures were subjected to Percoll gradient centrifugation to obtain highly purified parasites of specific stages. Giemsa-stained smears were microscopically observed over 2,000 RBCs to obtain differential counts.

Cultures (1% parasitaemia, 2% haematocrit) at each of the above mentioned stages were seeded in 96-well plates containing different concentrations of ΔFd and the plates incubated for 12 h (schizont), 24 h (trophozoite) and 48 h (ring) under standard culture condition. Smears were drawn, Giemsa-stained and analysed microscopically. Stage-specificity of action was assessed by observing the stage transitions in drug-treated samples against untreated controls.

### Cytotoxic activity of ΔFd on HeLa cells using MTT assay

The cytotoxic effects of ΔFd on mammalian cells was assessed by functional assay as described [31] using HeLa cells cultured in RPMI containing 10% fetal bovine serum, 0.21% sodium bicarbonate (Sigma) and 50 μg/mL gentamycin (complete medium). Briefly, cells (10^4^ cells/200 μl/well) were seeded into 96- well flat-bottom tissue culture plates in complete medium. Peptide solutions were added after 24 hr of seeding and incubated for 48 hr in a humidified atmosphere at 37°C and 5% CO_2_. DMSO (as positive inhibitor) was added at 10%. Twenty microlitres of a stock solution of MTT (5 mg/mL in 1X phosphate buffered saline) was added to each well, gently mixed and incubated for another 4 hr. After spinning the plate at 1500 rpm for 5 min, supernatant was removed and 100 μl of DMSO (stop agent) was added. Formation of formazon was read on a microtiter plate reader (Versa max tunable multi-well plate reader) at 570 nm. The 50% cytotoxic concentration (TC_50_) of drug was determined by analysis of dose–response curves.

### Haemolysis assay

Selectivity of haemolysis by peptides for infected erythrocytes (PRBC) *vs* uninfected erythrocytes (URBC) was examined by incubating the test molecules with URBCs and PRBCs respectively in phosphate-buffered saline (PBS). Briefly, fresh RBCs were spin washed (1600 RPM; 5 min) three times in PBS and re-suspended in PBS at 2% haematocrit. A 100 μl suspension was added to 96-well plate containing the peptides at different concentrations. PBS alone (for baseline values) and 0.4% Triton X-100 in PBS (for 100% haemolysis) were used as controls. After incubation at 37°C for 3 hr, the samples were centrifuged and supernatant was used to determine the haemolytic activity measured in terms of haemoglobin release as monitored by A_415_. Triton-treated control samples were diluted 10-fold before reading absorbance. Base line value (PBS control, <0.3% of the triton value) was subtracted from each data point. Percent haemolysis has been expressed with reference to 100% haemolysis value assigned to positive control (triton X 100 treated RBCs). To study haemolytic susceptibility of parasitized cells, URBCs were replaced with varying percentages (10% and 20%) of mixed stage parasitized cells. Three different control peptides (insulin prochitinase and E30, Table 1) were used to find whether there was specificity in the selective haemolytic action of ΔFd. Haemolysis of parasitized erythrocytes was also monitored microscopically by examining the Giemsa-stained smears. Parasitaemia of ΔFd treated and untreated cultures were microscopically counted for ring, trophozoite and schizont stages respectively. Susceptibility of different stages was compared with corresponding untreated control cultures.

### Fluorescence microscopic imaging

To probe mechanistic details of ΔFd-mediated, premature parasite egress, *P. falciparum* 3D7 culture (5% parasitaemia, 2% haematocrit) was incubated without (control) or with ΔFd (12.5 μM) at 37°C for 3 hr in a final volume of 100 μl made with RPMI (96 μl RPMI + 4 μl peptide solution).

Smears were processed as indicated: (a) flooded with 2% BSA/PBS (1 hr, 37°C), (b) washed and flooded (1 hr, 37^0^C) with monoclonal anti-band 3 antibody produced in mouse (Sigma) (1: 500 dilution in 1% BSA/PBS), (c) PBS washed and flooded with CY3 labelled anti-mouse antibody (Sigma)(1: 500 dilution in 1% BSA/PBS,1 hr*,* 37^0^C in dark), (d) PBS washed and flooded with DAPI (4, 6-diamidino-2-phenylindole) (invitogen) (500 ng/ml, 10 min, 37^0^C). After a final PBS wash the smears were observed under Nikon eclipse fluorescence microscope.

For studying peptide localization, *P. falciparum* cultures were individually incubated with FITC-ΔFd (2 μM), FITC-(ΔFm)_2_ (2 μM) or FITC-Insulin (3 μM) a) alone and b) together with DAPI in complete medium at 37°C for 30 min and the cells were spin washed (1,600 RPM, 5 min) twice with 1 X PBS to reduce background fluorescence. The cells were smeared on a glass slide, and fluorescence was visualized by using the respective filter settings for FITC and DAPI.

For studying the selectivity and route of transport of ΔFd into the red cell-resident malaria parasite, URBC and PRBC were incubated with FITC-ΔFd (4 μM) in parallel sets at 4°C *vs* at room temperature (25°C) for specified times and spin washed (1,600 RPM, 2 min) with complete medium (3 X 200 μl). The cells were smeared on a glass slide and both bright field images and fluorescence images (using FITC filter) were captured at 100 X magnification using Nikon eclipse fluorescence microscope. The software Adobe Photoshop was used to overlay the fluorescence image on the bright field image.

### Kinetics of peptide uptake

Kinetics of FITC-labelled peptide uptake was studied using Flow cytometer (BD FACS callibur). FITC- ΔFd (3 μM) was incubated for indicated time intervals with synchronized rings (~7% parasitaemia, 2% haematocrit) and synchronized trophozoites (~20% parasitaemia, 2% haematocrit) stage cultures in a total volume of 100 μl. Cells were spin washed (1 min) with 1 ml PBS and samples injected into FACS.

## Results

### Inhibition of *Plasmodium falciparum* growth by peptides

**Figure 1:**
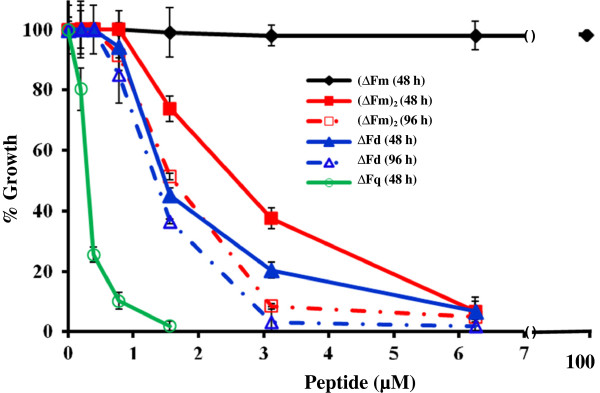
**Multivalent cationic, amphipathic helical peptides are potent inhibitors of the growth of malaria parasite in culture. **Dose-dependent effects of ΔFm (monomer), [(ΔFm)_2_ and ΔFd] (dimers) and ΔFq (quadrumer) on the growth of ring-stage synchronized *Plasmodium falciparum *(3D7) culture of malaria parasite. The anti-plasmodial potency increases in going from monomer ΔFm, to dimers [ΔFd, and (ΔFm)_2_] and the quadrumer ΔFq. The marginal difference in the comparative growth inhibition profiles of the two dimers at 48 hr *vs *96 hr suggests that there is predominantly early death. Each data point represents the mean+/− SD of three replicates.

The anti-plasmodial activities of the *de novo*-designed, synthetic peptides ΔFm, Fm, ΔFd, D-Lys-ΔFd, Fd, (ΔFm)_2_ and ΔFq (Table 1), were determined by quantitative SYBR Green I based estimation of DNA replication after one developmental cycle (48 hr) as a measure of growth (see Figure 1 for growth inhibition profiles of ΔFm, (ΔFm)_2_, ΔFd and ΔFq). In contrast to the monomers ΔFm/Fm (IC_50_ > 100 μM), the dimers showed potent {IC_50_ : (ΔFm)_2_ 2.4 μM, ΔFd 1.39 μM, D-Lys-ΔFd 1.8 μM, Fd 1.5 μM} dose dependent anti-plasmodial action against the growth of CQ-sensitive, blood stage parasite (*P. falciparum* 3D7) in culture. Interestingly the K-K_2_ branched tetrameric dendrimer ΔFq with IC_50_ 0.25 μM turned out to be the most potent anti-plasmodial in the present series. The progressive increment in anti-plasmodial potency with valency of the peptides suggests an oligomeric state of the peptide is associated with potency. The haemolysis-based selectivity indices for the potent peptides were >35 (ΔFd), >20 (ΔFm)_2_ , >27 (D-Lys-ΔFd), >33 (Fd) and 10 (ΔFq). The favourable index of >35 for ΔFd became the reason to study this peptide in greater detail. Further, (ΔFm)_2_ was studied since it was interesting to compare a linear dimer with a branched dimer. The K-K_2_ dendrimeric quadrumer was not studied in detail due to its poor haemolytic index. Several control peptides (Table 1) including sequences corresponding to prochitinase, E30, and bovine insulin showed no inhibition up to a concentration of 40 μM. Interestingly, both (ΔFm)_2_ and ΔFd retained their anti-plasmodial potencies also against the CQ-resistant Dd2 strain resulting in resistance index values of ~1 (Table 1). The more potent, branched dimer ΔFd showed IC_50_ value of 1.5 μM against the highly CQ-resistant INDO strain of *P. falciparum.* These results showed that dimerization potentiates anti-plasmodial activity by more than 50-fold over the corresponding monomer. The small but significant difference (student’s *T* test p: 0.013) between the potencies of the linear [IC_50_: (ΔFm)_2_ 2.4 μM] and branched (IC_50_: ΔFd 1.39 μM) dimers suggests that the mode of dimerization may also play a subtle role in modulation of potency. When examined for comparative potency in 48 hr (one cycle) *vs* 96 hr (two cycles) assays, only marginal increments in potency [1.5 fold (ΔFm)_2,_ and 1.1 fold ΔFd] were observed at 96 hr (Figure 1).

### Ring *vs* trophozoite: selectivity in the action of ΔFd

**Figure 2:**
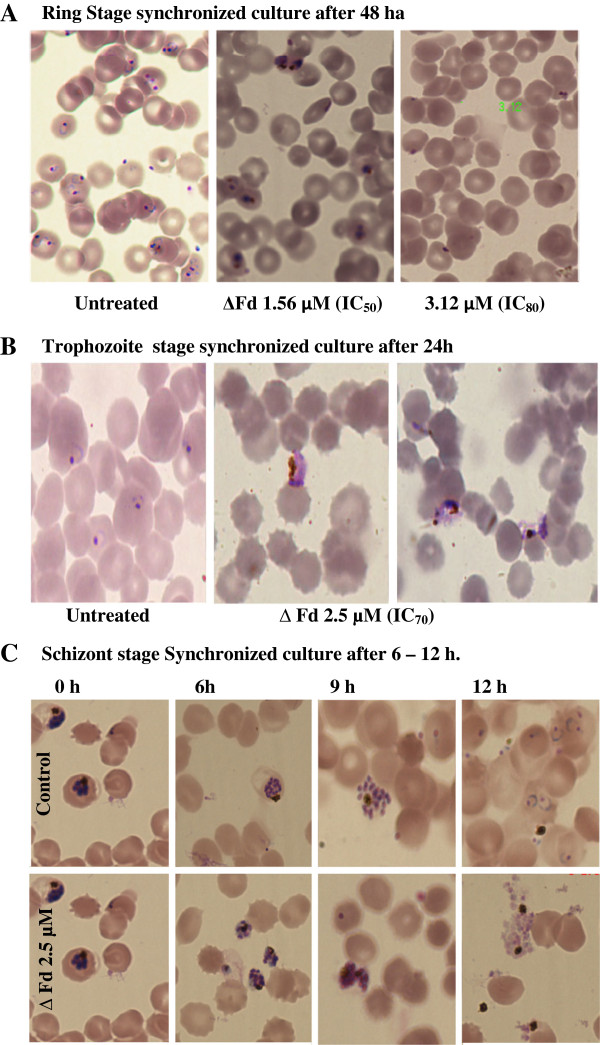
**Microscopy of anti-plasmodial action of ΔFd on *Plasmodium falciparum *3D7. (A) **Untreated or ΔFd-treated, ring-stage synchronized cultures (parasitaemia 1%) were observed after 48 hr. Untreated culture shows high ring-stage parasitaemia, ΔFd IC_50_ and IC_80_ treated cultures show low trophozoite-stage and low ring-stage arrested parasitaemia respectively, (**B**) Untreated or ΔFd-treated trophozoite-stage synchronized cultures were observed after 24 hr. Untreated culture shows intracellular rings while the ΔFd-treated culture shows anomalously egressed trophozoites. Note the selectivity in action on parasitized cells with no effect on uninfected cells. **(C)** Untreated or ΔFd-treated schizont stage synchronized cultures were observed at 6–12 hr. While schizonts with the characteristic rosette arrangement of merozoites are intracellular at 6 hr in untreated culture, they have (a) prematurely egressed and (b) lost the rosette arrangement of merozoites in the peptide treated culture (For zoom of the images, see additional file 10, panel A). At 12 hr while the merozoites in control have invaded fresh red cells to form rings, the merozoites of peptide treated cultures have failed to invade and form rings (For quantitative account of decrease in invasion events , see additional file 10, panel C).

In order to find whether there was ring *vs* trophozoite selectivity in the action of ΔFd, microscopic evaluation of its action was studied against parasitized red cells synchronized at ring (Figure 2A) and trophozoite (Figure 2B) stages respectively. When the ring stage parasite culture was treated with IC_50_ dose of ΔFd, it was observed (Figure 2A) that, after 48 hr of culture, the rings had progressed only up to the trophozoite stage suggesting biochemical arrest and the resulting interception of the progression to the schizont stage. Further at IC_80_, it was observed that the rings did not mature even to the trophozoite stage and the arrest of the parasite cycle was at the ring stage. Since ΔFd at its IC_50_ caused arrest at trophozoite stage (Figure 2A), the effect of ΔFd at its IC_70_ (2.5 μM) was tested on cultures synchronized at trophozoite stage. It was interesting to see (Figure 2B) that the peptide caused anomalous egress of trophozoites. Even as 95% of trophozoites were found to be extracellular (Additional file 8); this phenomenon was not a consequence of non-specific haemolysis since uninfected red cells were not affected (Figure 2B). Thus it appears that at ~ IC_80_ rings are metabolically arrested and the RBCs harbouring them are not lysed while such doses cause selective lysis of parasitized RBCs that harbour trophozoites. The observation of MSP3 staining in ~ 40% of the anomalously egressed trophozoites (Additional file 9) is worth noting.

### ΔFd is fairly non toxic to mammalian HeLa cells

**Figure 3:**
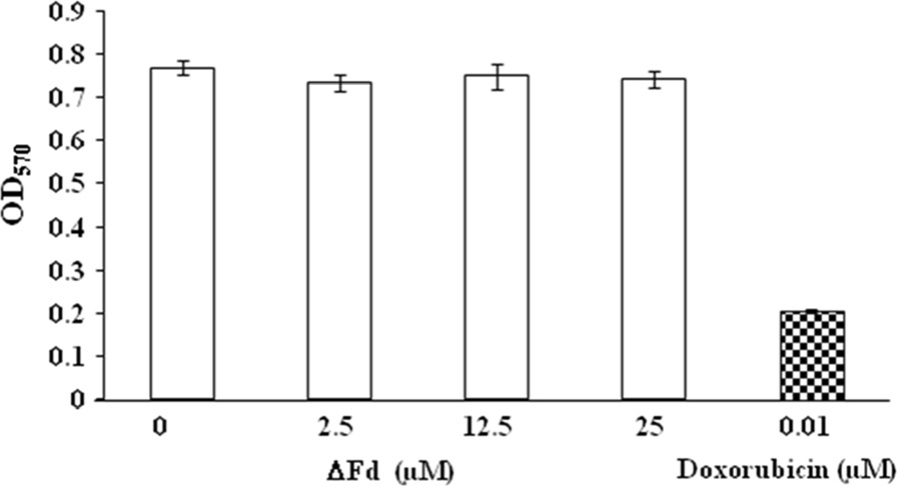
**Histogram showing results of MTT assay measuring viability of HeLa cells incubated with ΔFd at different concentrations. **Data shows mean and standard deviation of three independent observations.

Toxicity of ΔFd to mammalian cells was examined by MTT assay. HeLa cells incubated with varying concentrations (2.5-25 μM) of ΔFd (Figure 3) did not show any toxicity. This suggests that the therapeutic index (TC_50_ Mammalian cells/IC_50_*P.falciparum*) of this peptide (>16) is promising.

### ΔFd causes premature egress of undifferentiated Schizonts

While it is unnatural for trophozoites to egress, the egress of schizonts is a natural process that leads to increased parasitemia. It was therefore interesting to find the effect of ΔFd on egress of schizonts. As shown (Figure 2C), the peptide treated cultures showed premature egress at 6 h at a time when the schizonts in the control culture were intracellular. A close look at the schizonts of the control and the peptide treated cultures (see Additional file 10, panel A) revealed that (a) The characteristic symmetric rosette arrangement of merozoites seen in control at 6 hr and 9 hr is absent in the prematurely egressed schizonts of the peptide treated culture and (b) the well differentiated merozoites of the control are invasion competent which enables them to form new rings while the merozoites of the peptide-treated culture are invasion disabled resulting in no new infections of red blood cells. Interestingly merozoites from both the control and peptide treated schizonts were found to be MSP3+ (Additional file 9).

### Selective haemolytic effect of ΔFd

**Figure 4:**
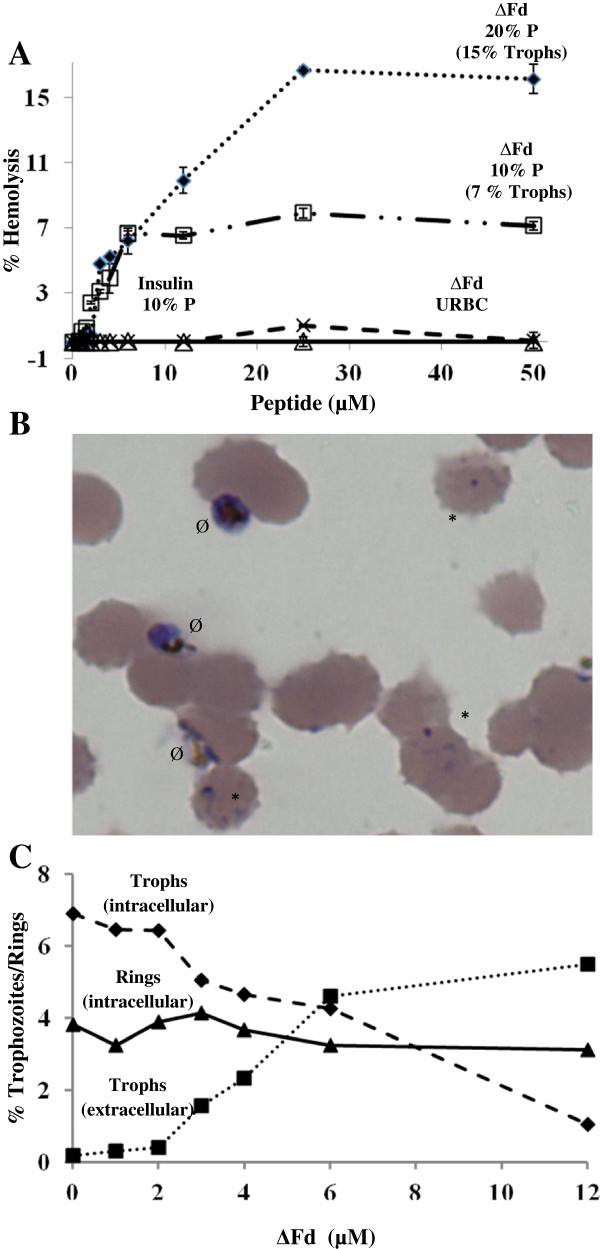
**ΔFd causes selective haemolysis of parasitized red cells leading to anomalous egress of trophozoites. (A)** Samples of mixed stage parasite culture at different parasitaemia (P) (% figures on respective curves) were incubated (3 hr) with the indicated concentrations of peptide and percentage haemolysis estimated by A_415_. Control peptide (insulin) with infected red cells (10% trophozoite-stage parasitaemia, solid line); ΔFd with URBC, (dashed line); ΔFd with 10% parasitaemia (rings 3%, trophozoites 7%, dashed dotted line); and ΔFd with 20% parasitaemia (rings 5%, trophozoites 15%, dotted line). Two other control peptides (prochitinase and E30) behaved like insulin (data not shown). **(B)** Shows microscopic analysis of the selective sensitivity of trophozoites (Ø, anomalously egressed) *vs *rings (*, intracellular) at 12 μM ΔFd, **(C)** shows dose-dependent selective effect of ΔFd on anomalous egress of trophozoites but not rings, monitored microscopically after incubation (3 hr). Data shown were obtained after counting 2,000 erythrocytes.

The anomalous egress of trophozoites via haemolysis motivated an examination of the selectivity in the action of ΔFd against parasitized **(**PRBC) *vs* uninfected red cells (URBC) over a range of peptide concentrations. As shown (Figure 4A), while the URBCs showed considerable resistance to lysis, the PRBCs showed increasing lysis both with increasing concentration of peptide and also with increasing parasitaemia. It may be noted that the observed lysis is proportional to the percentage of trophozoites in the cultures tested. Thus in the two mixed cultures shown in Figure 4A, the percentage haemolysis values of 7% and 16% correspond to percentage trophozoite populations of ~ 7% and 15%, respectively. Further microscopic evaluation of mixed parasite cultures treated with ΔFd (12 μM) revealed (Figure 4B) that only the trophozoites and not the rings were observed to be extracellular. In order to find if the observed lysis of infected cells was specific to ΔFd or would any peptide in general cause similar lysis, three control peptides (insulin, E30 and prochitinase) (Figure 4A) were tested and found not to show any haemolysis up to a concentration of 40 μM. Microscopic examination of ~ 2,000 cells from infected red cell cultures revealed a peptide concentration dependent inverse relation between intracellular *vs* extracellular trophozoites (Figure 4C). Also evident from this figure is the stability of ring-infected cells up to 12 μM of **Δ**Fd.

### Anomalously egressed trophozoites are not surrounded by host cell membrane

**Figure 5:**
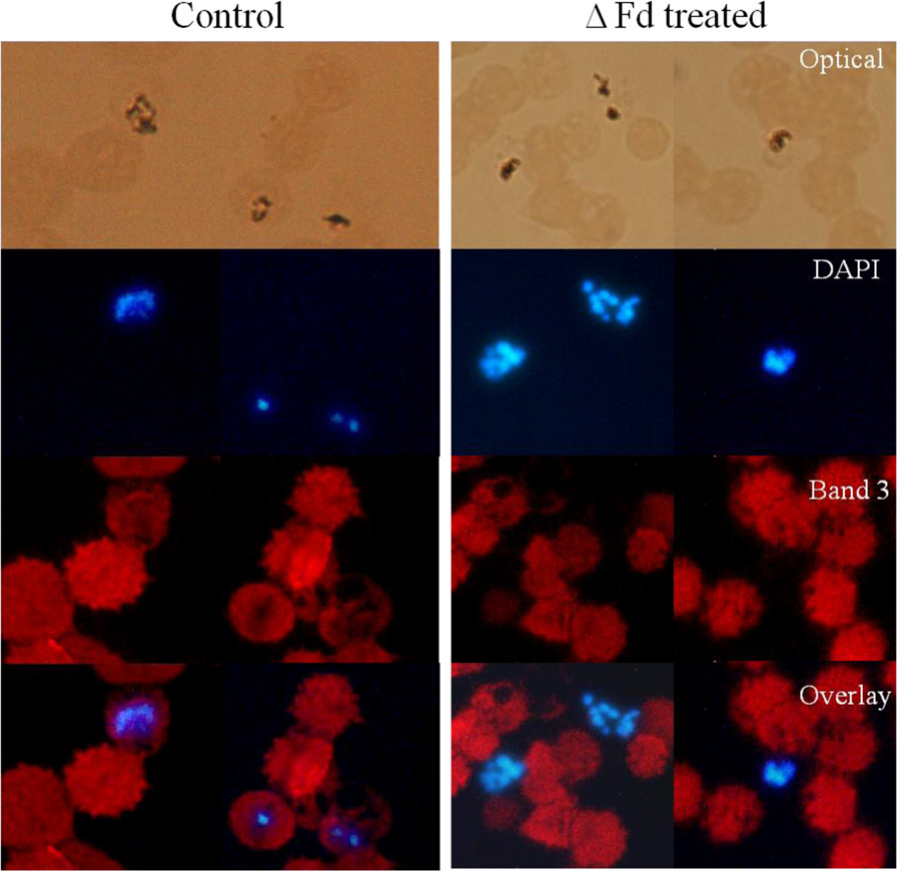
**ΔFd-mediated parasite egressed from red blood cells are not coated with host cell membrane. **Bright field optical images (top panel) show haemozoin crystals that are intracellular in control and appear to be extracellular in ΔFd (12.5 μM)-treated sample. Panel 2 shows DAPI stained nuclei of the malaria parasite. Panel 3 (immunostaining with band 3 antibody) indicates that band 3 (red) was seen in all cells. Panel 4 (overlay of DAPI and band 3) indicates that the parasites (staining blue) in control panel are intracellular and flanked by band 3 stain. But the ΔFd-treated parasites, which egressed anomalously, are extracellular and not flanked by band 3.

Immunostaining with band 3 antibody was done in order to find if the trophozoites egressed in response to **Δ**Fd were free or packaged in host cell membrane. As shown in Figure 5, while the untreated culture showed the DAPI stained parasites to be intracellular and flanked by band 3 staining (red), the egressed extracellular trophozoites in the **Δ**Fd-treated culture (12.5 μM) had no band 3 staining around them. This observation suggests that egress does not involve host cell membrane and is likely to be mediated via lysis of the host cell.

### Dimers ΔFd and (ΔFm)_2_ show selective penetration into *Plasmodium*-infected RBCs

**Figure 6:**
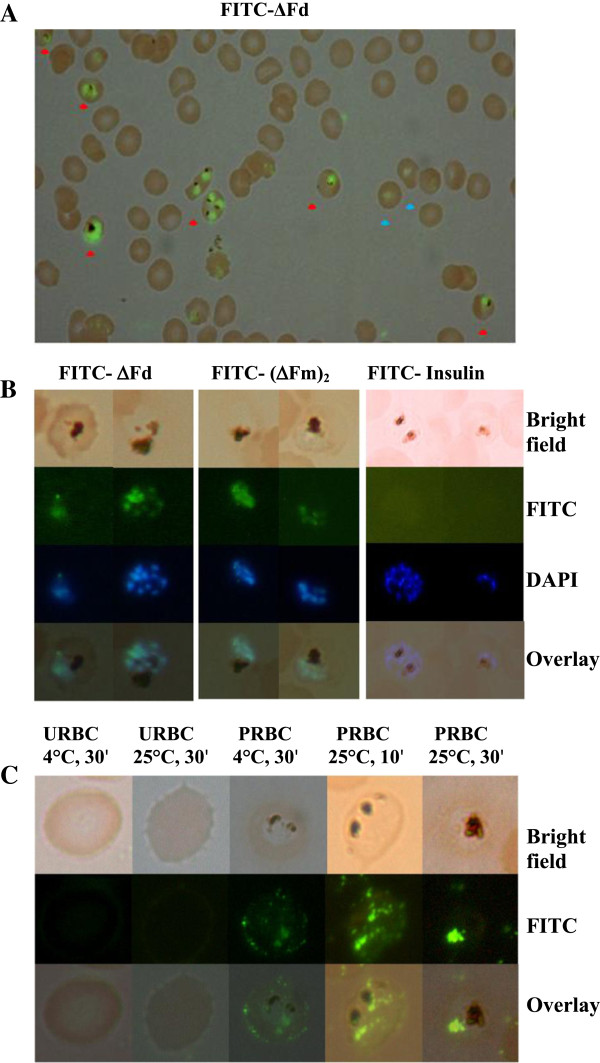
**Cellular localization of FITC-labelled peptides in *Plasmodium falciparum *-infected red blood cells. **(**A**) FITC-ΔFd (3 μM) was incubated with parasitized culture (30 min, 37°C). Fluorescence image overlaid on optical image shows that FITC-ΔFd exhibits selective entry into parasitized RBCs. Arrow heads*: *red (trophozoites showing haemozoin), blue (likely to be ring stages with low fluorescence). (**B**) FITC-ΔFd and FITC- (ΔFm)_2_ but not FITC-insulin are internalized by PRBC. The overlay of FITC fluorescence (green) with DAPI (blue) suggests that these two peptides bind to DNA of the parasite. (**C**)Transport of FITC-ΔFd (4 μM) from RBC surface to parasite: ΔFd has selective affinity for infected RBC (PRBC) surface. The slow entry of FITC-ΔFd into PRBC at 4°C becomes fast at 25°C.

To gain a better understanding of the anti-plasmodial action of the two dimeric peptides, localization of peptides was studied using fluorophore-labelled peptides. Fluorescence microscopy with FITC-labelled **Δ**Fd showed that this peptide was selective in targeting the parasite inside the infected RBC (Figure 6A). Co-localization of FITC florescence (green) with DAPI florescence (blue) (Figure 6B) indicated that the two peptides bind to the DNA of the malaria parasite. In order to check whether entry of the two dimers was specific or would any other peptide also enter parasitized cells, FITC-labelled insulin was examined for uptake by the parasitized cells. Fluorescence microscopy revealed that there was no accumulation of FITC-insulin in parasitized cells suggesting specificity in uptake and anti-plasmodial action of the two dimeric peptides. Since no fluorescence was observed on the red cell surface even as there was intense fluorescence intracellularly, it was surmised that the uptake of the peptide may be faster than the time (30 min) given for the experiment. In order to capture early events in transfer of the peptide from the RBC surface to the parasite, a comparative uptake study at 4°C *vs* at 25°C was performed. As shown (Figure 6C), while the URBC showed no staining, the PRBC at 4°C showed predominantly surface staining with a modicum of intracellular staining. However PRBC at 25°C showed a transition from surface to intracellular staining at 10 min which became completely intracellular at 30 min. Thus it appears that parasite-infected red blood cells are well geared for rapid uptake of this peptide.

### Uptake kinetics of ΔFd

**Figure 7:**
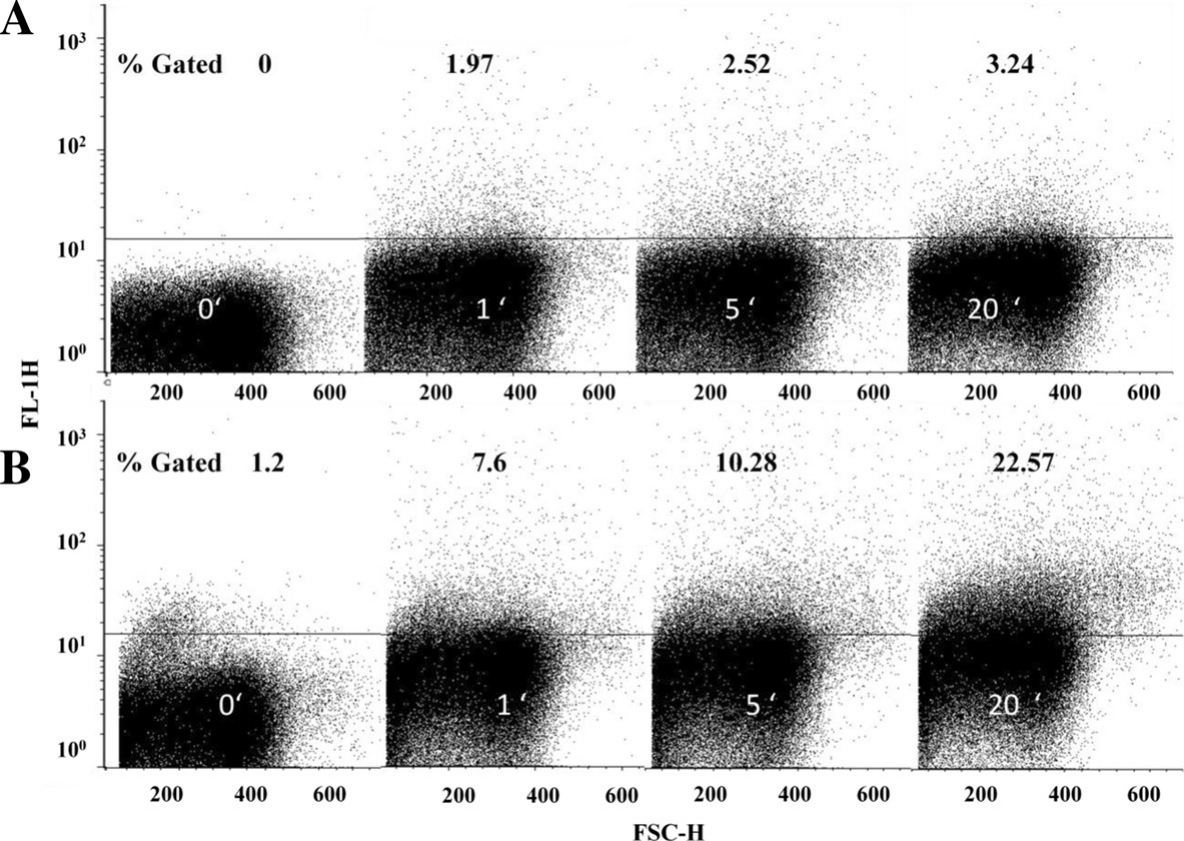
**Kinetics of ΔFd entry into parasitized red blood cells (synchronized rings (~7% parasitaemia) and synchronized trophozoites (~20% parasitaemia). **Uptake of fluorescently tagged FITC-ΔFd (2.5 μM) was monitored by flow cytometry at 25°C. Panels A and B depict the peptide uptake profiles obtained with rings and trophozoites, respectively. Zero minute profiles correspond to samples not treated with peptide. Figures against percentage gated indicate the number of cells stained above the threshold line. Note (a) the fast uptake and progressive increase in the number of fluorescent signals with time, and (b) faster uptake by trophozoites compared to ring-stage parasitized cells.

In order to assess the kinetics of uptake of the fluorescently tagged peptide into the infected red cells, a time-dependent analysis of the phenomenon was studied by FACS. Monitoring the uptake in ring-synchronized cultures (Figure 7) revealed a low uptake (1.97%) at the first minute rising to ~3% at 20 min. In contrast to rings, the analogous peptide uptake by trophozoites was found to be fast at the very first minute (7.6%) with further substantial rise to 22% at 20 min.

## Discussion

The success of antibiotics is based upon the characteristic molecular targets that distinguish the prokaryotic bacteria from the nucleated eukaryotic cells [32,33]. Cationic, amphipathic helical, antibiotic peptides also seem to gain specificity by exploiting the fact that bacteria have a preponderance of anionic lipids, such as phosphatidylglycerol and bis(phosphatidyl)glycerol (cardiolipin), conferring a negative charge on their surface. In contrast, their eukaryotic counterparts have a high density of zwitterionic lipids such as phosphatidylcholine and phosphatidylethanolamine, enabling their surfaces to be largely neutral [34,35]. A well-studied and yet curious feature of the human red blood cell is the transition from FITC-Annexin negative to FITC-Annexin positive status upon infection with the malaria parasite [14]. It is well known that this phenomenon is caused by the translocation of the anionic phosphatidylserine from the inner to the outer leaflet of the lipid bilayer. Thus infection with *Plasmodium* confers an anionic character to the red blood cell giving it a shade of semblance to a bacterial membrane. Focusing on the altered membrane asymmetry seen in the infected red cell, the interesting anti-plasmodial properties of several *de novo*-designed, cationic, amphipathic, helical, bonafide membrane-active anti-bacterial peptides have been examined in the present studies.

The first observation of the comparative anti-plasmodial potencies of two monomeric (ΔFm, Fm) and four dimeric peptides {ΔFd, Fd, D-Lys-ΔFd, (ΔFm)_2_}(Table 1) indicated that the dimers (IC_50_ 1.39- 2.4 μM) were about two orders of magnitude more potent than the monomers (IC_50_ >100 μM). Among the dimeric peptides {ΔFd: IC_50_ 1.39 μM, D-Lys-ΔFd: IC_50_ 1.8 μM, (ΔFm)_2_: IC_50_ 2.4 μM, Fd: IC_50_ 1.5 μM}, the lysine-branched ΔFd was chosen for detailed mechanistic studies since it had favourable features of anti-plasmodial potency and selectivity index (>35). Interestingly, in going from this bivalent-branched dimer ΔFd to the tetravalent K-K_2_-branched quadrumer ΔFq (IC_50_: 0.25 μM), a further six-fold potentiation was observed. However, as shown in Table 1, this potentiation was associated with a decline in selectivity index from >35 (ΔFd) to 10 (ΔFq). Nevertheless, the trend of increasing anti-plasmodial potency with increasing valency (monomer to dimer to quadrumer) of the peptide suggests that oligomerization on cell surfaces may play an important role in the anti-plasmodial action of these cationic, amphipathic peptides. It is important to note that crystal structures of several ΔF-containing peptides have revealed the propensity of this planar aromatic residue to engage in long-range, multicentred interactions (N-H…O, C-H…O, C-H…π, and N-H…π) that can stabilize oligomeric states like the ΔF zipper [36] in the absence of linker, or the helical hairpins in the presence of appropriate linker [37,38]. The coming together of optimal values of anti-plasmodial potency and selectivity indices (haemolytic selectivity index >35 and mammalian cell cytotoxicity index >16) in the lysine-branched dimeric ΔFd became the motivation to unravel mechanistic details of the anti-plasmodial action of this peptide. (ΔFm)_2,_ the corresponding linear dimer, was also studied in some experiments to explore if the mode of dimerization may influence the anti-plasmodial actions of these two dimeric peptides.

The essentiality of apicoplast, an organelle of cyano-bacterial origin in the malaria parasite, is well known to make the parasite vulnerable to antibiotics,such as tetracycline, clindamycin and thiostrepton [39,40], which are known to cause delayed death in malaria parasite. This phenomenon, caused by targeting of the apicoplast or the mitochondrion, is characterized by a significantly lower IC_50_ post second cycle (at 96 hr) *vs* the first cycle (at 48 hr) [41]. In order to find if the anti-plasmodial peptides under study may be targeting organelles such as the apicoplast of the malaria parasite, comparative anti-plasmodial potencies against *P. falciparum* 3D7 were determined at both 48 hr and 96 hr. Since the data (Figure 1) did not show a significant reduction of IC_50_ at 96 hr, the possibility that ΔFd and (ΔFm)_2_ may cause early death by influencing several other targets besides the apicoplast and the mitochondria cannot be ruled out.

In trying to gain a better understanding of the probable mechanisms that confer the malaria parasite growth inhibitory properties on the dimeric peptide ΔFd, the peptide-treated samples were examined by microscopy. As shown (Figure 2A), in comparison to the untreated control (high ring-stage parasitaemia), while the IC_50_-treated ring stage synchronized culture was found to have the initial low parasitaemia (1%) and growth arrest at trophozoite stage, the IC_80_ treated sample was found to have the intial parasitaemia (1%) with the few parasitized cells showing arrested, probably dead pyknotic ring forms. Interestingly, the microscopic examination of the ΔFd-treated, trophozoite-enriched culture (Figure 2B) showed the presence of extra erythrocytic Giemsa-positive trophozoites alongside uninfected red blood cells. Indeed manual counting of a large number of fields (Additional file 8) indicated that over 95% of the trophozoites were in fact extracellular. The presence of extracellular trophozoites in the midst of intact uninfected red blood cells was suggestive of the selective haemolytic action of ΔFd on parasitized cells causing anomalous release of trophozoites following 24-hr incubation.

The transition of ring stage to trophozoite stage in presence of ΔFd at its IC_50,_ indicates that while this low dose is sufficient to arrest trophozoites, it is clearly not sufficient to halt the ring from moving to the trophozoite stage (Figure 2A). This heightened sensitivity of trophozoite-stage cultures over ring-stage cultures may be related to the enormous red cell reorganizational changes associated with a fast feeding, actively metabolizing and replicating life style of trophozoite in comparison with the more sedentary ring stage. The selective lysis of trophozoite-bearing cells (Figure 4B) also suggests that such remodelling of the trophozoite harbouring red cell membrane [42] may be rendering it more vulnerable to the action of membrane active peptides like the ΔFd. The greater vulnerability of trophozoite bearing over ring-bearing red cells is evident also from the fact that peptide-mediated haemolysis is directly proportional to the percentage trophozoites in mixed stage culture samples (Figure 4A).

Trophozoite egress, induced by the peptide, is not natural to the life cycle of the malaria parasite. Hence, it was important to find if host cell membranes-may be involved in the process. To address this issue, the peptide-treated sample was exposed to immunostaining with band 3 antibody. As shown (Figure 5), the egressed trophozoites were not flanked by band 3 staining suggesting that the process is more likely to be caused by lysis of the infected host cell. A closer examination of the phenomenon of ΔFd-mediated anomalous egress of trophozoites revealed that in trophozoite-ring mixed culture exposed to FITC-ΔFd at low concentrations (2 μM) and short time (30 min) (Figure 5A), the peptide seems to enter and attack the parasite from within without causing immediate lysis of the infected red cell. However when trophozoite-stage culture was exposed to ΔFd for longer times (24 hr), at similar low concentrations (2 μM), this peptide seemed to cause selective lysis of parasitized cells leading to anomalous egress of trophozoites (Figure 2B). Further, at higher concentrations (12.0 μM) this peptide caused selective lysis of red cells harbouring trophozoites within 3 hr (Figure 4B).

**Figure 8:**
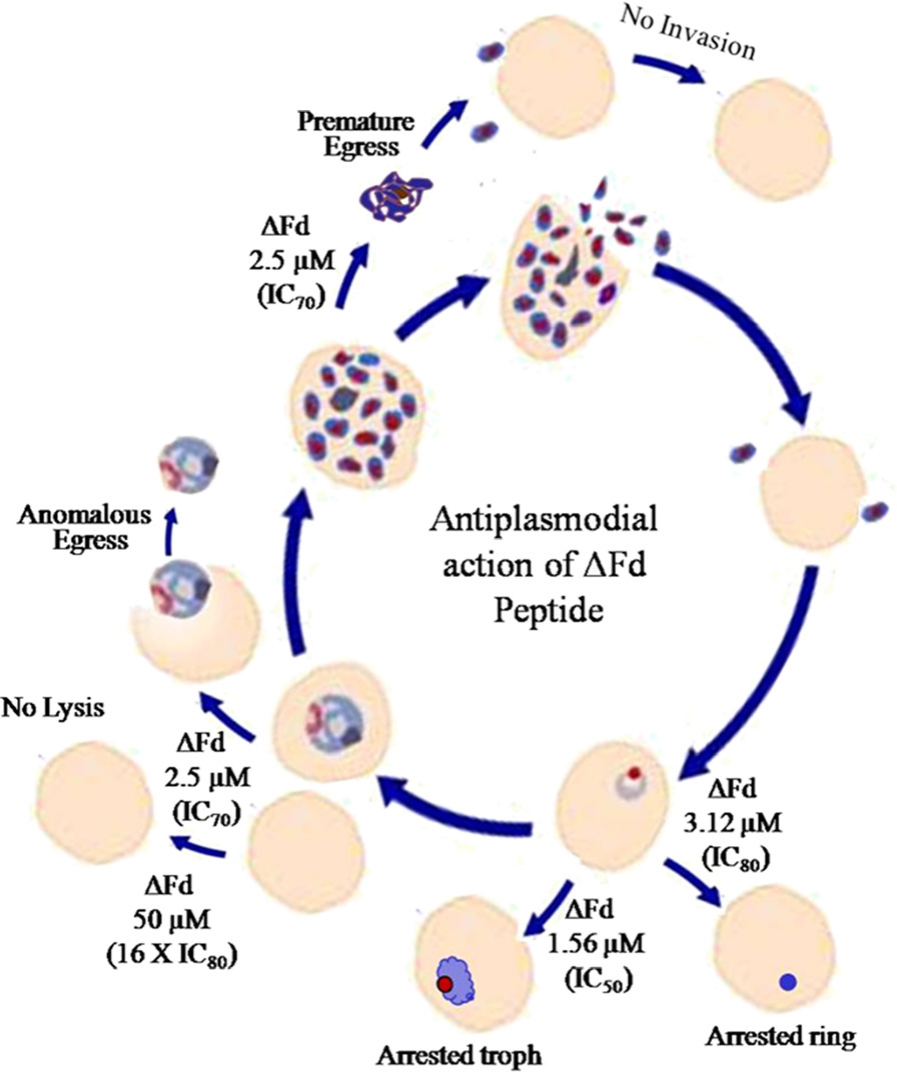
**Model of antiplasmodial action of ΔFd. ΔFd causes growth arrest of rings, anomalous egress of trophozoites and premature egress of schizonts. **Its IC_80_ (3.12 μM) and IC_50_ (1.56 μM) cause arrest of rings and trophozoites respectively and its IC_70_ (2.5 μM) causes the anomalous egress of trophozoites and premature egress of schizonts. In both cases the parasite fails to proliferate since egressed trophozoites cannot differentiate into schizonts and the premature, undifferentiated egressed schizonts seem to release merozoites that are invasion incompetent. The peptide shows good selectivity against parasitized RBCs since 16X IC_80_ fails to lyse healthy RBCs.

Unlike trophozoites, schizonts have an intrinsic program of egress that causes the release of numerous merozoites leading to infection of fresh red cells causing amplification of infection and increasing the severity of disease. Hence it was interesting to find if ΔFd may perturb the programmed process of egress in schizonts. As shown (Figure 2C and Additional file 10), this peptide caused premature egress of schizonts. As a consequence, the egressed schizonts which showed lumps of amplified DNA did not exhibit the characteristic symmetrically organized rosette appearance of merozoites seen in the untreated control schizont. Further the merozoites from the peptide treated culture showed a significantly low invasion efficiency in comparison to the control merozoites (Additional file 10). Figure 8 summarizes the versatility of ΔFd to target each stage of the life cycle of *P. falciparum* in characteristic and decisive ways with good selectivity.

Malaria parasites go to extraordinary means to modify RBC membrane, which separates them from the external world. These modifications include a marked increase in erythrocyte membrane fluidity [43-46], alterations in host cell lipid fatty acid composition [47,48] and phospholipid-transbilayer distribution [49], enhancement of the rate of lipid transbilayer movement [50,51] and increased permeability through newly formed pores on the erythrocyte membranes [52,53]. As a part of these major re-organizational events, the malaria-infected red cell is well known to exhibit a translocation of the anionic phosphatidylserine from the inner leaflet to the outer leaflet of the bi-layer [13,54]. This more negative cell surface may provide the force for the fast and specific uptake of cationic peptides by the malaria-infected red cell. Previous studies have indicated that high levels of cellular uptake can be achieved through the inclusion of cationic residues into arginine-based peptide oligomers [55]. The positive molecular charge facilitates charge-driven uptake through the plasma membrane, which exhibits a potential gradient that can electrophorese cationic species from the extracellular space into the cell [56,57]. Interestingly, a recent study has demonstrated that membrane asymmetry can be altered and maintained in the altered state by externally added poly-L-lysine [58]. The combined microscopic (Figure 6) and FACS analysis (Figure 7) suggests that ΔFd enters the infected cells and stains rings and trophozoites within a few minutes. Thus it is quite likely that ΔFd and (ΔFm)_2_, the two cationic dimeric peptides studied here, in close resemblance to poly-L-lysine, may first home on those infected red blood cells that show slightly more anionic character as a result of alterations in membrane asymmetry and binding of these cationic peptides could further enhance and maintain this anionic character facilitating the stronger binding and faster internalization of peptides into the infected cells.

The ability of ΔFd to cross the host red cell membrane, the parasitophorous vacuole membrane, the parasite plasma membrane and also the parasite nuclear membrane to reach the nucleus of the parasite (Figure 6B), indicates its resemblance to cell-penetrating peptides which are known to have a lipophilic-cationic character. Even as the peptide was apparently targeting the DNA of the parasite, the absence of FITC-ΔFd on the host red cell membrane or all the subsequent membranes mentioned above was puzzling. It was surmised that these localizations may have been missed due to the rapidity of the process of peptide uptake. In order to capture some stages preceding the intranuclear entry of the peptide, the peptide-staining experiment was performed as a function of both time and temperature. As shown (Figure 6C), the images captured at 4°C (30 min) indeed showed predominant staining on the host cell surface. In contrast, the images corresponding to 25°C (10 min) and 25°C (30 min) showed progressively greater staining of the intracellular parasite nuclear material. These results suggest that this peptide crosses several membranes of the infected red cells before entering the nucleus.

The most probable reasons for the significantly enhanced potency of the dimers ΔFd/Fd over the monomers ΔFm/Fm include increased membrane binding and permeabilization, enhanced binding affinity for DNA and proteins and enhanced biochemical stability against degradation by proteases. These properties originating from increased avidity and affinity of interactions unique to dimeric peptides and absent in monomeric peptides have been described previously [25]. In studies on the antibiotic action of these peptides it has previously been observed that the requirements of helicity for potent antibiotic action are much higher for the gram positive *Staphylococcus.aureus* than is the case with the Gram-negative *Escherichia coli*. In contrast, as shown in the present study, all dimers {(ΔFd, Fd, D-Lys-ΔFd, (ΔFm)_2_} are nearly equipotent against *P. falciparum* (Table 1). This suggests that different conformational and topological properties of peptides may be important for their activity against different organisms.

Some important features of these peptides as drugs against malaria include their favourable resistance indices (Table 1) that allow them to rapidly kill both drug-sensitive and drug-resistant strains of malaria parasite with equal potencies, their amphipathic nature that gives them drug-like character, and their ability to permeabilize and penetrate biological membranes, which allows them to attack target cells both from the surface as well as intracellularly. In addition, the presence of the conformationally constrained, non-protein, amino acid didehydrophenylalanine in both ΔFd and (ΔFm)_2_ provides considerable protection against proteolytic degradation [25]. Even as these two dimeric peptides offer similar profiles of anti-plasmodial actions, a judicious choice for further improvisation should be the branched dimer ΔFd over the linear dimer (ΔFm)_2_ since (a) the former is little more potent against *P. falciparum*, (b) the branched dimer is more stable against proteases [25], and (c) the branched dimer has better economics of production since the time it takes to synthesize a branched dimer is half as much as the time it takes to assemble a linear dimer.

## Conclusion

This study reports the anti-plasmodial action of ΔFd, a *de novo*-designed, cationic, lysine-branched amphipathic, helical peptide. *In vitro* assays suggest good selectivity (>35), good resistance index (1.1) and low mamamalian cell cytotoxicity, as a promise of ΔFd against malaria. The strategy adopted by ΔFd to inhibit the growth of malaria parasite appears to be broadly two-fold: (a) involving growth arrest without causing lysis of red cell (at IC_50_-IC_100_), and (b) anomalous egress of trophozoites and premature egress of undifferentiated schizonts leading to death of the parasite (at > IC_100_).

## Abbreviations

CQ: Chloroquine; ΔF: Didehydrophenylalanine; ΔFm: ΔF containing monomeric decapeptide; ΔFd: Lysine branched dimer of ΔFm; (ΔFm)2: Linear dimer of ΔFm; DAPI: 4',6-diamidino-2-phenylindole; FACS: Fluorescence activated cell sorter; FITC: Fluorescein isothiocyanate; P. falciparum: Plasmodium falciparum; IC100: Inhibitory concentration causing 100% inhibition of growth; PRBC: Parasitized red blood cell; URBC: Uninfected red blood cell; Troph: Trophozoite; ΔFq: The K-K2dendrimer presenting a quadrumer form of ΔFm.

## Competing interests

The authors declare that they have no competing interests.

## Authors’ contributions

NKK and JS carried out the experiments to determine the antiplasmodial potencies of different peptides, NKK performed mechanistic experiments including FACS and immunofluorescence microscopy, DS conceived of the study, participated in its design, coordination and brain storming and drafted the manuscript. All authors read and approved the final manuscript.

## Supplementary Material

Additional file 1 RPHPLC profiles of control peptides. Click here for file

Additional file 2 RPHPLC profiles of ΔFm and ΔFd. Click here for file

Additional file 3 ESMS of RPHPLC purified prochitinase, E30 and Insulin. Click here for file

Additional file 4 ESMS of RPHPLC purified ΔFm and ΔFd. Click here for file

Additional file 5 MALDI mass spectrum (Bruker Daltonics Flex analysis) of RPHPLC purified linear dimeric peptide. Click here for file

Additional file 6 Chromatographic and mass spectral characterization of ΔFq. Click here for file

Additional file 7 ESMS of RPHPLC purified Fm, Fd and D-Lys-ΔFd. Click here for file

Additional file 8 Microscopic differential counts of ΔFd (2.5 μM) treated trophozoites after 24 h. Click here for file

Additional file 9 ΔFd treated schizonts express MSP3. Click here for file

Additional file 10 ΔFd causes premature egress of schizonts. Click here for file
