# Supplementary figures and images for: Anti-plasmodial action of de novo-designed, cationic, lysine-branched, amphipathic, helical peptides
Source: Malar J. 2012 Aug 1;11:256. doi: 10.1186/1475-2875-11-256 (PMC3502156; doi:10.1186/1475-2875-11-256)

Additional File 1. RPHPLC profiles of Control peptides

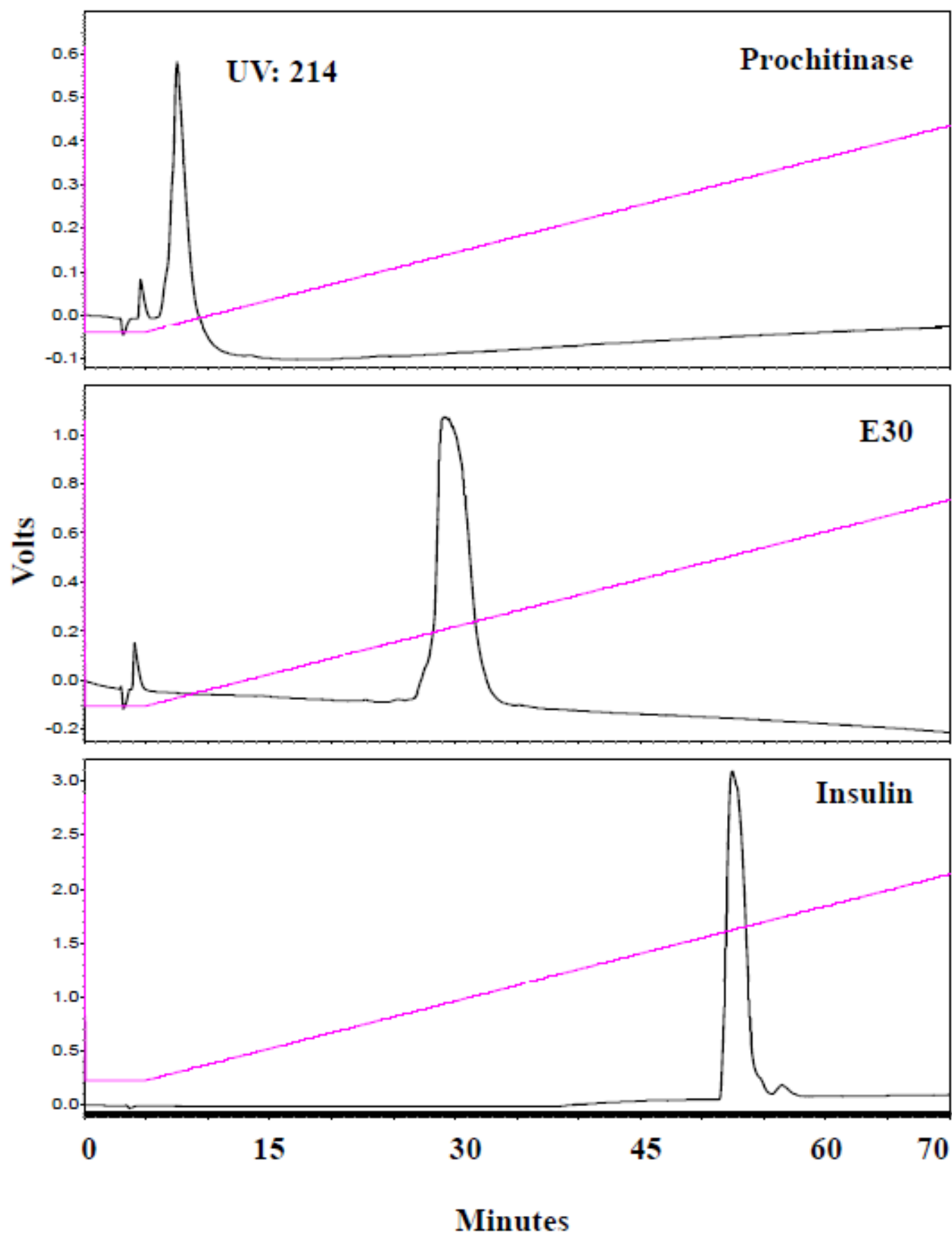

Supplement: Additional file 1 — RPHPLC profiles of control peptides. [file 1475-2875-11-256-S1.pdf]

Additional File 2. RPHPLC profiles of  $\Delta Fm$  and  $\Delta Fd$

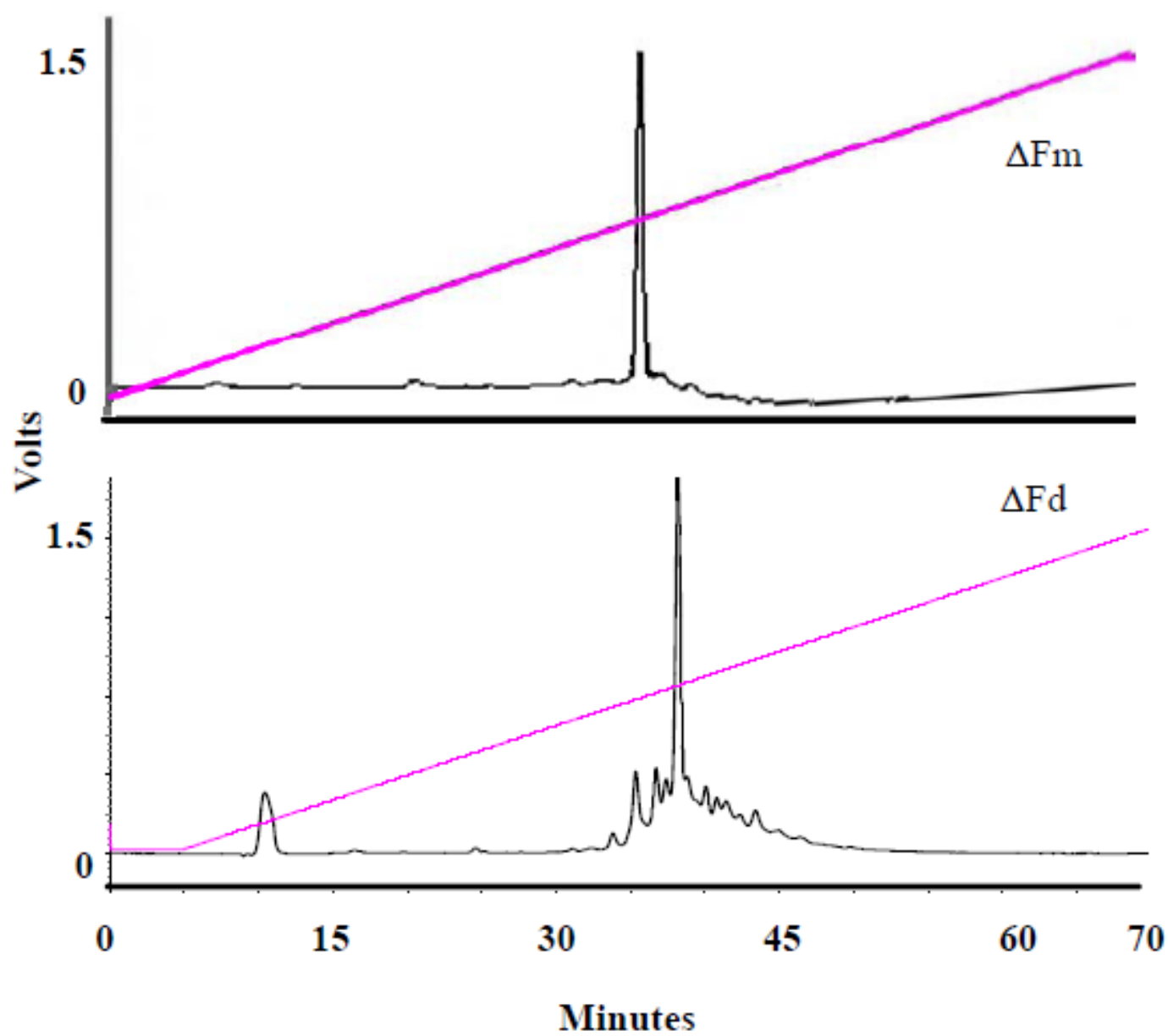

Supplement: Additional file 2 — RPHPLC profiles of ΔFm and ΔFd. [file 1475-2875-11-256-S2.pdf]

Additional File 3. ESI mass spectra of RPHPLC purified peptides

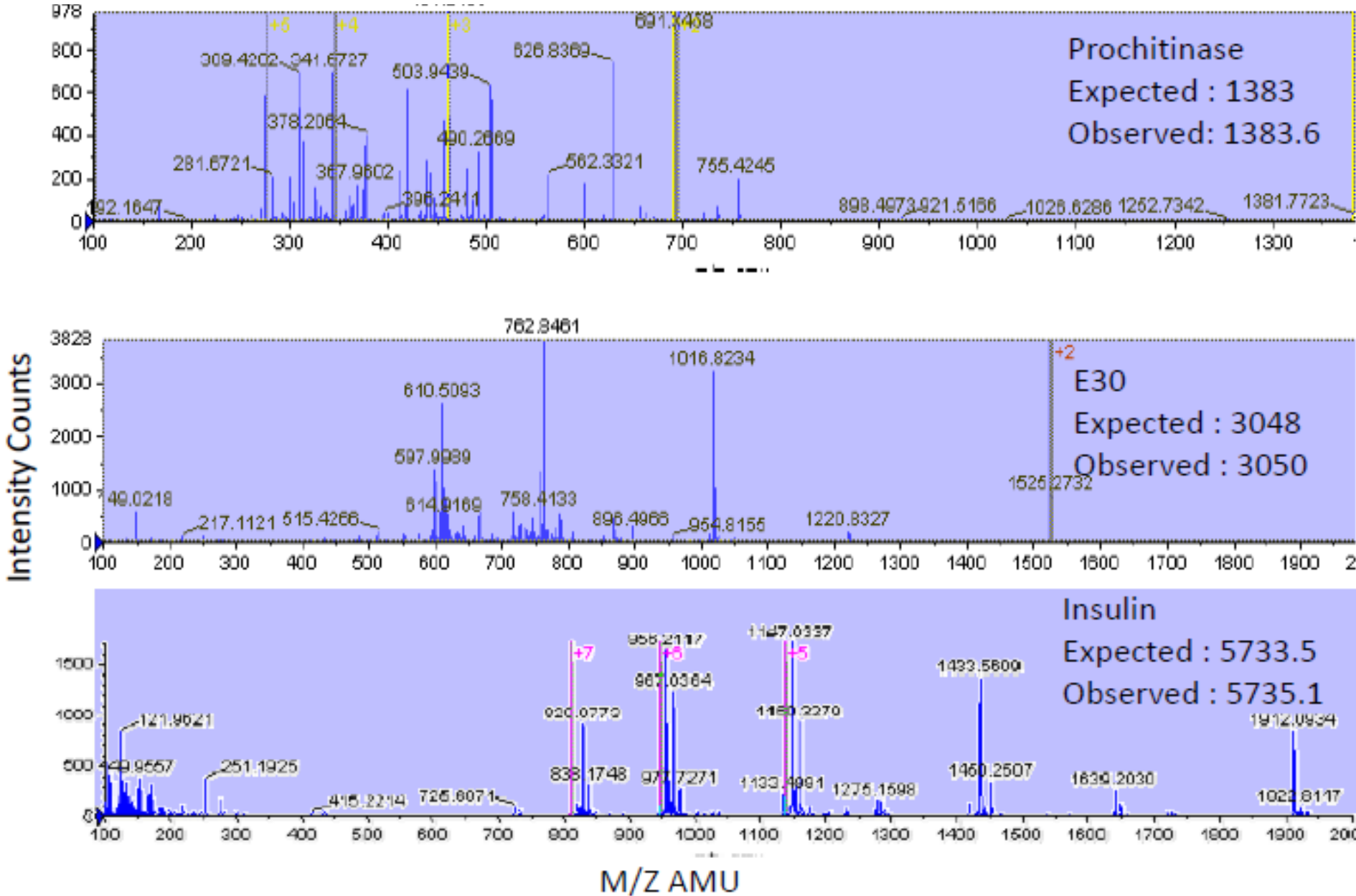

Supplement: Additional file 3 — ESMS of RPHPLC purified prochitinase, E30 and Insulin. [file 1475-2875-11-256-S3.pdf]

Additional File 4. ESI mass spectra of RPHPLC purified peptides

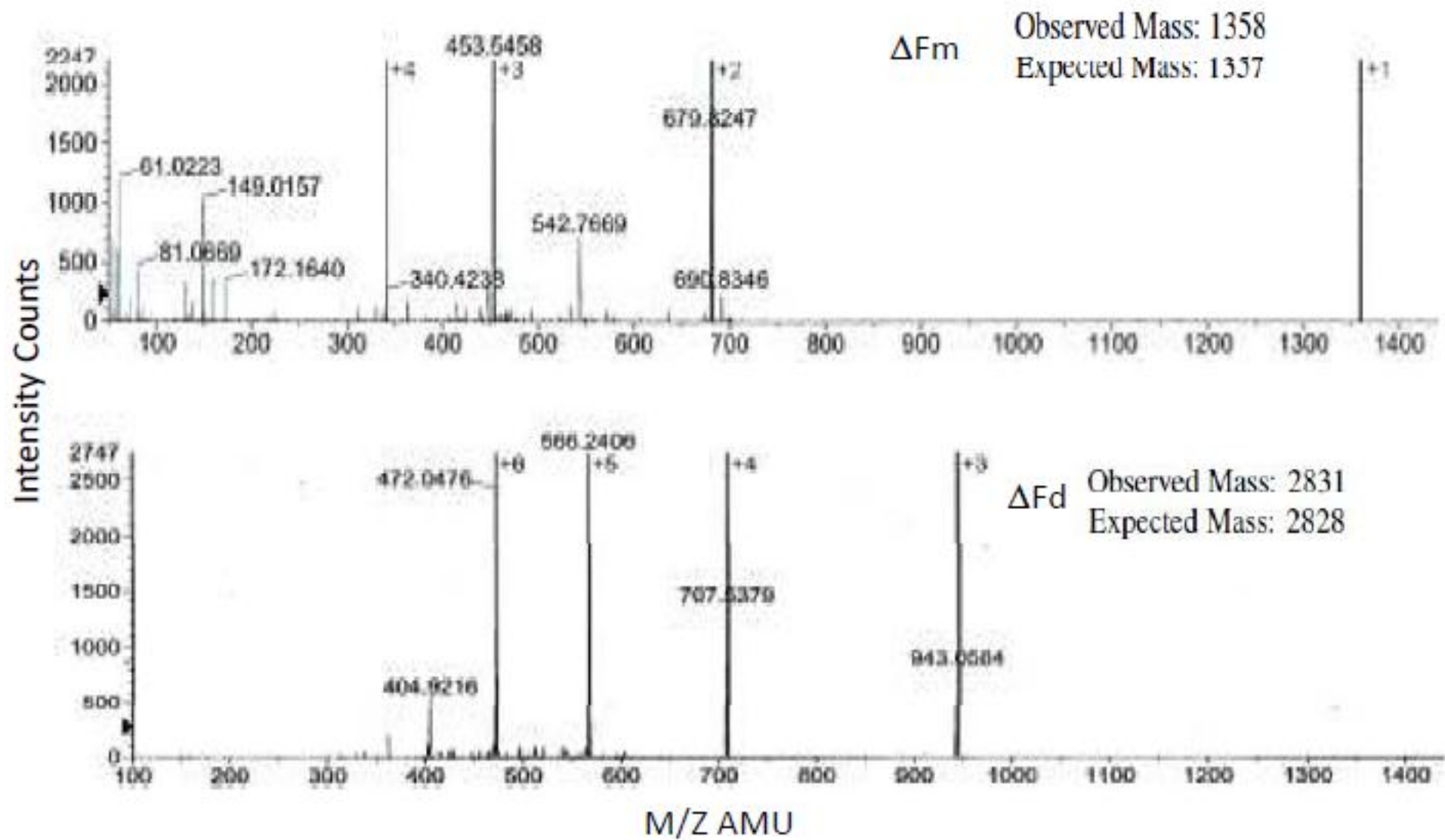

Supplement: Additional file 4 — ESMS of RPHPLC purified ΔFm and ΔFd. [file 1475-2875-11-256-S4.pdf]

Additional File 7. ESI mass spectra of RPHPLC purified peptides

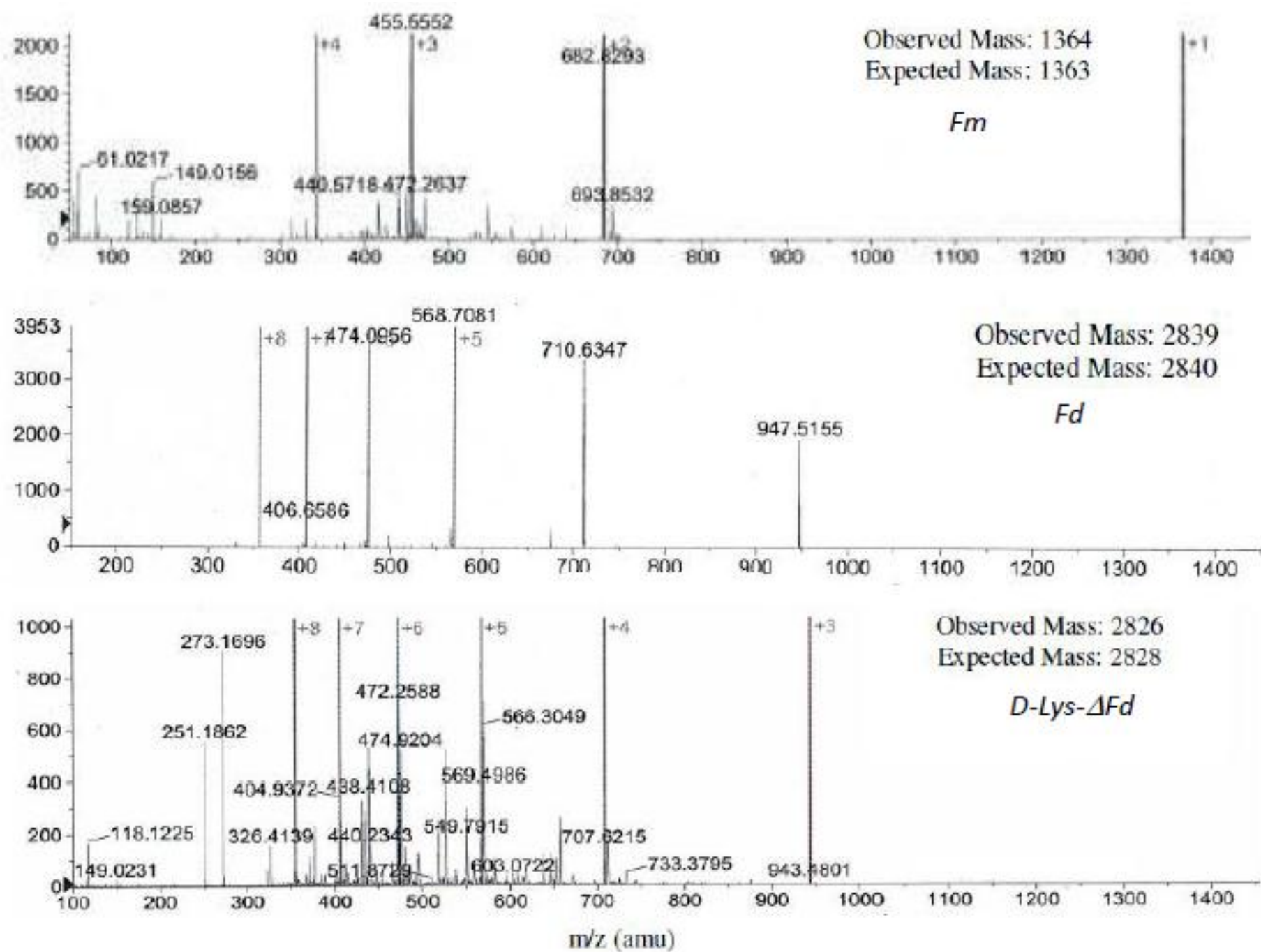

Supplement: Additional file 7 — ESMS of RPHPLC purified Fm, Fd and D-Lys-ΔFd. [file 1475-2875-11-256-S7.pdf]
