# Supplementary material for: Anti-plasmodial action of de novo-designed, cationic, lysine-branched, amphipathic, helical peptides
Source: Malar J. 2012 Aug 1;11:256. doi: 10.1186/1475-2875-11-256 (PMC3502156; doi:10.1186/1475-2875-11-256)

Additional File 5. MALDI mass spectrum (Bruker Daltonics Flex analysis) of RPHPLC purified linear dimeric peptide.

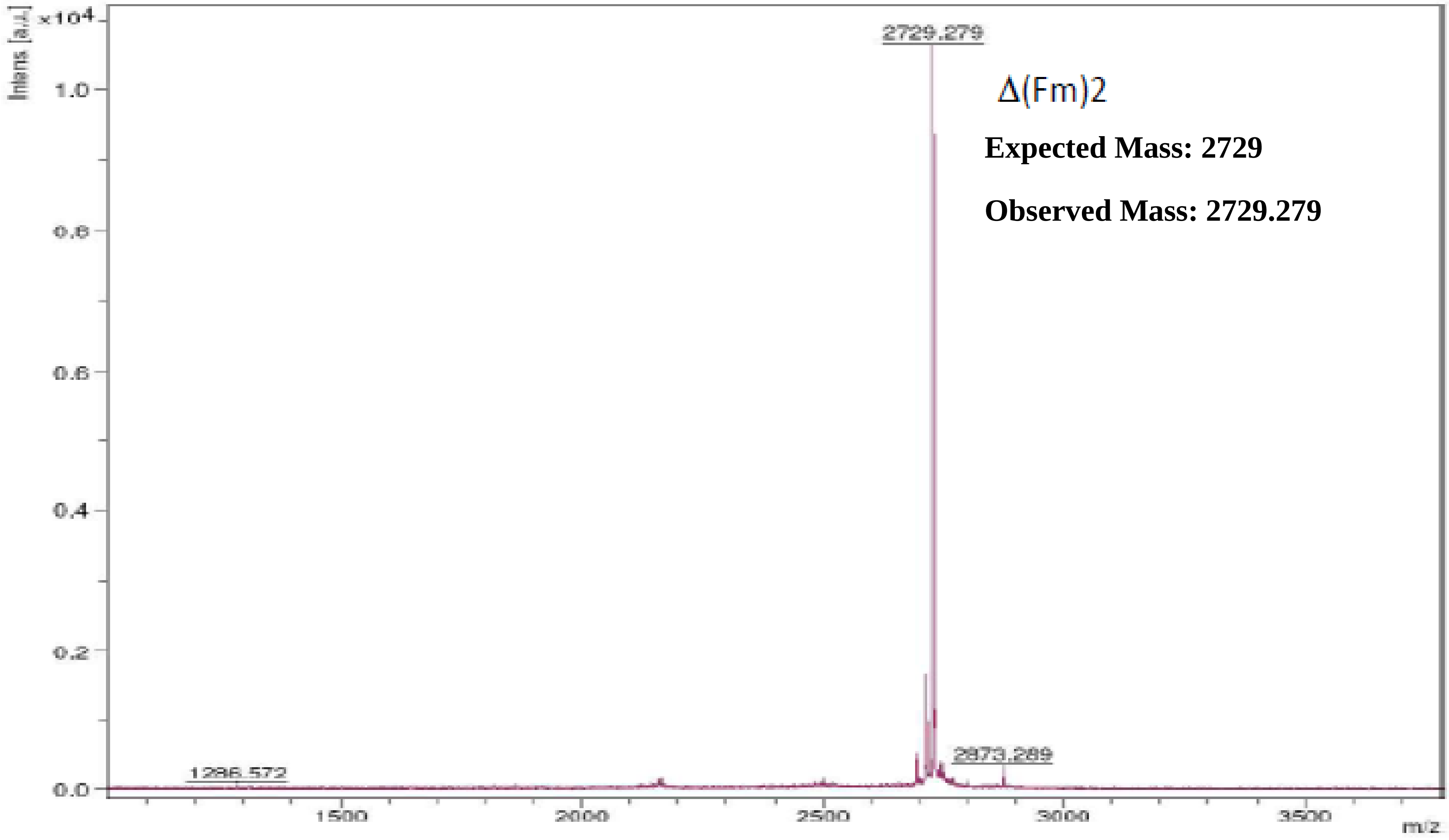

Supplement: Additional file 5 — MALDI mass spectrum (Bruker Daltonics Flex analysis) of RPHPLC purified linear dimeric peptide. [file 1475-2875-11-256-S5.pdf]
