# Supplementary material for: Anti-plasmodial action of de novo-designed, cationic, lysine-branched, amphipathic, helical peptides
Source: Malar J. 2012 Aug 1;11:256. doi: 10.1186/1475-2875-11-256 (PMC3502156; doi:10.1186/1475-2875-11-256)

Additional File 6. Chromatographic and mass spectral characterization of ΔFq

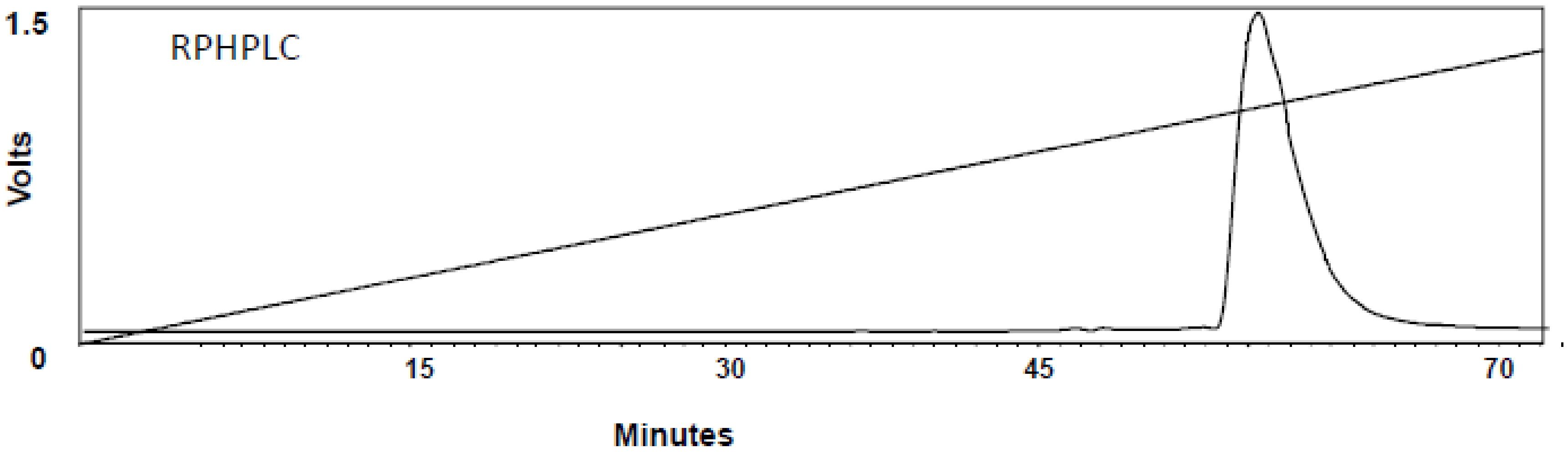

Expected Mass: 5765 Da

Observed Mass: 5774.82 Da

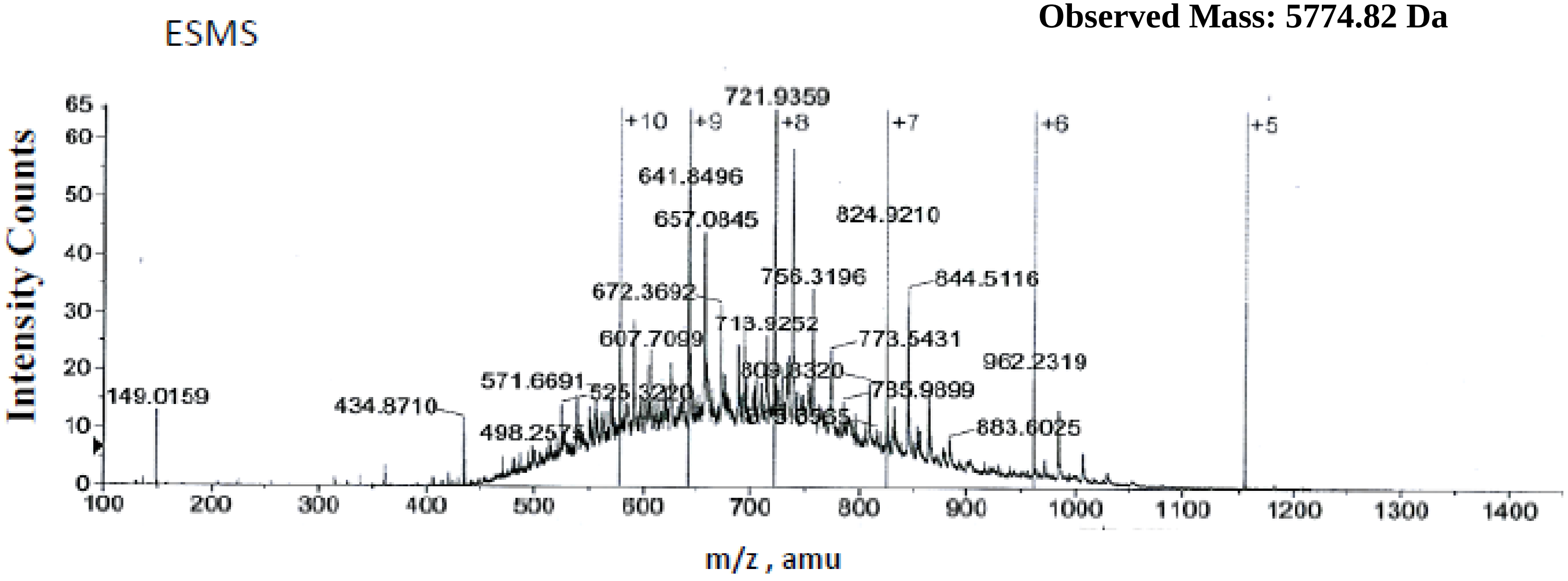

Supplement: Additional file 6 — Chromatographic and mass spectral characterization of ΔFq. [file 1475-2875-11-256-S6.pdf]
