# Supplementary material for: Anti-plasmodial action of de novo-designed, cationic, lysine-branched, amphipathic, helical peptides
Source: Malar J. 2012 Aug 1;11:256. doi: 10.1186/1475-2875-11-256 (PMC3502156; doi:10.1186/1475-2875-11-256)
Supplement: Additional file 8 — Microscopic differential counts of ΔFd (2.5 μM) treated trophozoites after 24 h. [file 1475-2875-11-256-S8.pdf]

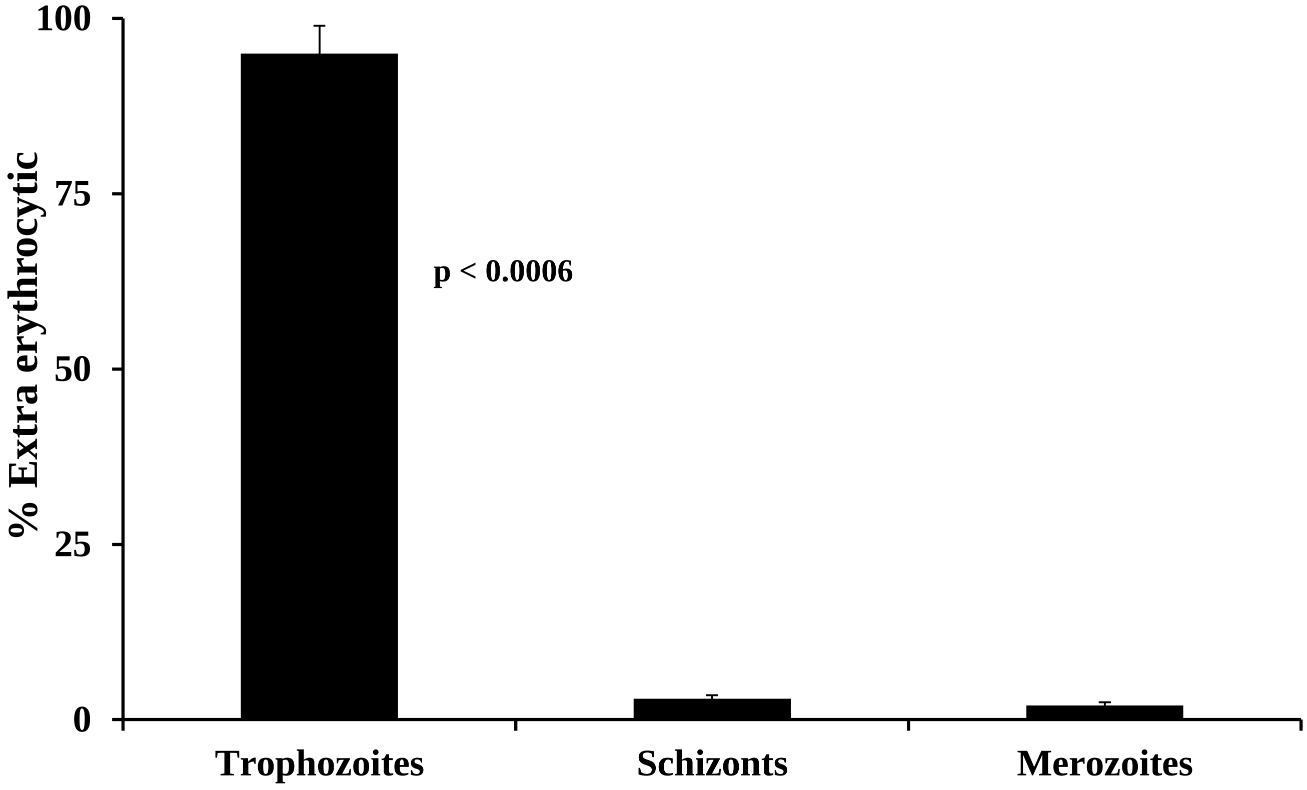

Additional File 8. Microscopic differential counts of ΔFd (2.5 µM) treated trophozoites after 24 h. Counting of 2,000 red cells shows that >95% trophozoites are extracellular. Under similar condition the untreated control showed rings with no extracellular parasites. Each bar represent the mean ± standard deviation of three independent observations. p value obtained from student's T test denotes the significantly higher number of extracellular trophozoites in comparison with untreated control.
