# Supplementary material for: Anti-plasmodial action of de novo-designed, cationic, lysine-branched, amphipathic, helical peptides
Source: Malar J. 2012 Aug 1;11:256. doi: 10.1186/1475-2875-11-256 (PMC3502156; doi:10.1186/1475-2875-11-256)
Supplement: Additional file 9 — ΔFd treated schizonts express MSP3. [file 1475-2875-11-256-S9.pdf]

Additional File 9.

Method

Immunohistochemical Fluorescence staining of MSP3 in ΔFd treated Trophozoite and Schizont cultures.

To probe mechanistic details of ΔFd-mediated, premature parasite egress, *P. falciparum* 3D7 culture (2% parasitaemia, 2% haematocrit) was incubated without (control) or with ΔFd (2.5 µM) at 37°C for 6 hr in a final volume of 100 µl made with RPMI (96 µl RPMI + 4 µl peptide solution). Smears were processed as follows: (a) flooded with 2% BSA/PBS (1 hr, 37°C), (b) PBS washed and (c) flooded (1 hr, 37^0C) with polyclonal anti-MSP3 antibody produced in mouse (1: 100 dilution in 1% BSA/PBS), (d) PBS washed and (e) flooded with alexa fluor 488 labelled anti-mouse antibody (Sigma) (1: 500 dilution in 1% BSA/PBS, 1 hr, 37^0C in dark), (e) PBS washed and (f) flooded with DAPI (4, 6-diamidino-2-phenylindole) (invitrogen) (500 ng/ml, 10 min, 37^0 C). After a final PBS wash the smears were observed under Nikon eclipse fluorescence microscope using appropriate filters.

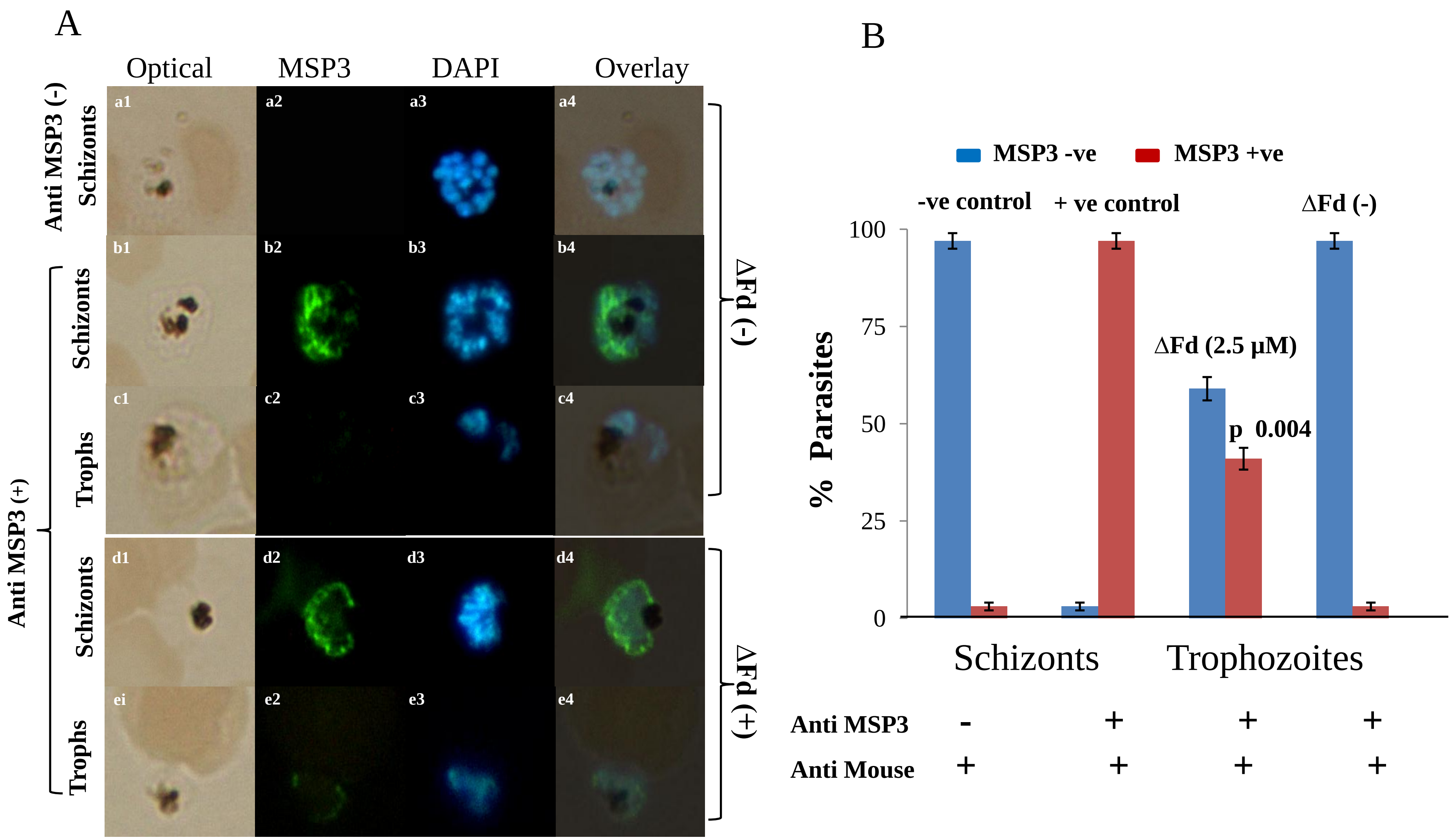

Additional File 9. ΔFd treated Schizonts express MSP3. Panel A: Smears of synchronized schizont and trophozoite stage cultures were prepared for staining with anti MSP3 followed by DAPI staining. In the absence of anti MSP3 antibody, schizonts showed no fluorescence (a2) while strong green fluorescence was observed when the smears were preincubated with anti MSP3 antibody (b2). The control intraerythrocytic trophozoites were negative for MSP3 (c2). However upon treatment with peptide some of the trophozoites exhibited weak MSP3 staining (e2). Panel B shows a) status of % MSP3 staining in negative (- anti MSP3) and positive (+ anti MSP3) schizont controls obtained after observing 700 RBCs and b) faint MSP3 staining was observed on ~ 40% of trophozoites. . Each bar represent the mean standard deviation three independent observations. p value is based on MSP3+ trophozoites in peptide treated versus untreated cultures.
