# Supplementary material for: Anti-plasmodial action of de novo-designed, cationic, lysine-branched, amphipathic, helical peptides
Source: Malar J. 2012 Aug 1;11:256. doi: 10.1186/1475-2875-11-256 (PMC3502156; doi:10.1186/1475-2875-11-256)
Supplement: Additional file 10 — ΔFd causes premature egress of schizonts. [file 1475-2875-11-256-S10.pdf]

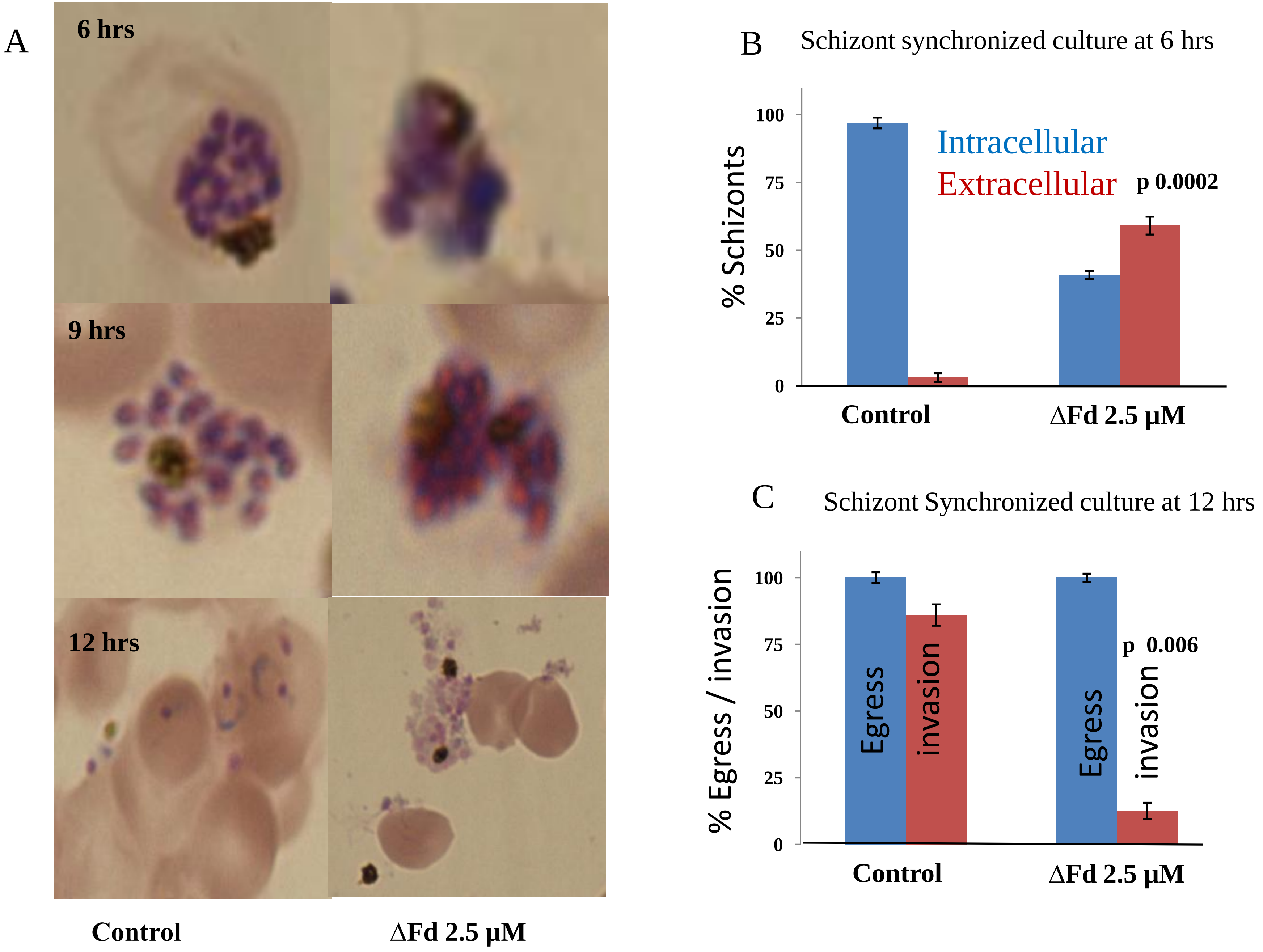

Additional File 10.

Additional File 10. ΔFd causes premature egress of Schizonts. Panel A shows that while the control schizont is intracellular at 6 hrs, the peptide treated prematurely egressed schizont has lost the characteristic symmetrical rosette pattern of merozoites seen in control schizonts. At 12 hrs, in contrast to the control culture (showing well formed rings), the peptide treated culture is showing merozoites that have failed to invade. Panel B shows that at 6 hrs ~60 % schizonts treated with ΔFd (2.5 µM) are extracelluar against a meagre 2 % egress seen in control. Panel C shows that most merozoites in control were invasion competent while most merozoites in the peptide treated sample were invasion incompetent. 2000 RBCs were counted to obtain the data shown. Each bar represent the mean standard deviation of three independent observations. Student`s T test p values are based on extracellular schizonts (panel B) and invasion events (panel C).
